# Triplet State Radical Chemistry: Significance of the Reaction of ^3^SO_2_ with HCOOH and HNO_3_

**DOI:** 10.1021/jacs.4c03938

**Published:** 2024-05-09

**Authors:** Josep M. Anglada, Marilia T. C. Martins-Costa, Joseph S. Francisco, Manuel F. Ruiz-López

**Affiliations:** † Departament de Química Biològica (IQAC − CSIC), c/Jordi Girona 18, Barcelona E-08034, Spain; ‡ Laboratoire de Physique et Chimie Théoriques, UMR CNRS 7019, University of Lorraine, CNRS, BP 70239, Vandoeuvre-lès-Nancy 54506, France; § Department of Earth and Environmental Science and Department of Chemistry, University of Pennsylvania, Philadelphia, Pennsylvania 19104-6316, United States

## Abstract

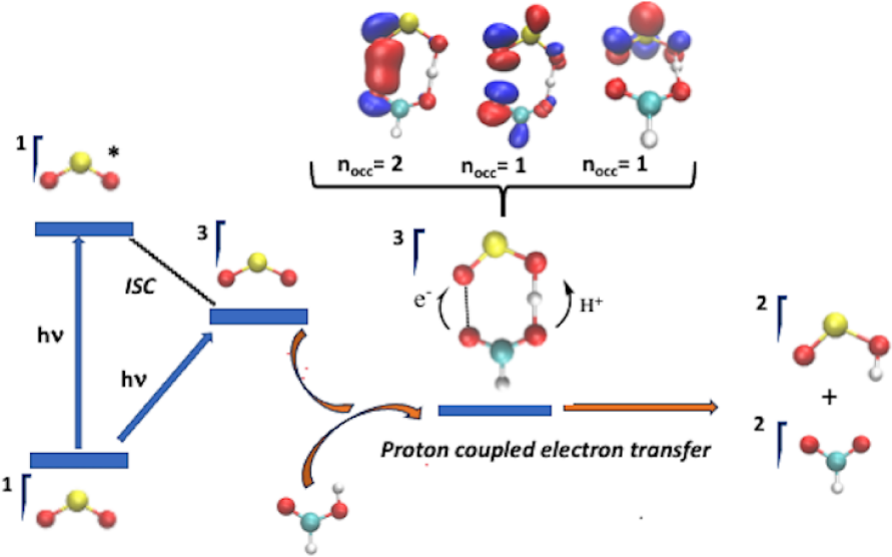

The triplet excited states of sulfur dioxide can be accessed in the UV region and have a lifetime large enough that they can react with atmospheric trace gases. In this work, we report high level ab initio calculations for the reaction of the a^3^B_1_ and b^3^A_2_ excited states of SO_2_ with weak and strong acidic species such as HCOOH and HNO_3_, aimed to extend the chemistry reported in previous studies with nonacidic H atoms (water and alkanes). The reactions investigated in this work are very versatile and follow different kinds of mechanisms, namely, proton-coupled electron transfer (*pcet*) and conventional hydrogen atom transfer (*hat*) mechanisms. The study provides new insights into a general and very important class of excited-state-promoted reactions, opening up interesting chemical perspectives for technological applications of photoinduced H-transfer reactions. It also reveals that atmospheric triplet chemistry is more significant than previously thought.

## Introduction

The chemistry of radicals is among the most important processes in nature, playing an important role in the chemistry of the atmosphere, in materials science, in organic synthesis, and in biological processes.^1,2^ In particular, the abstraction of one hydrogen atom by a radical, eq 1, has special relevance.
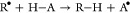
1

**Scheme 1:**
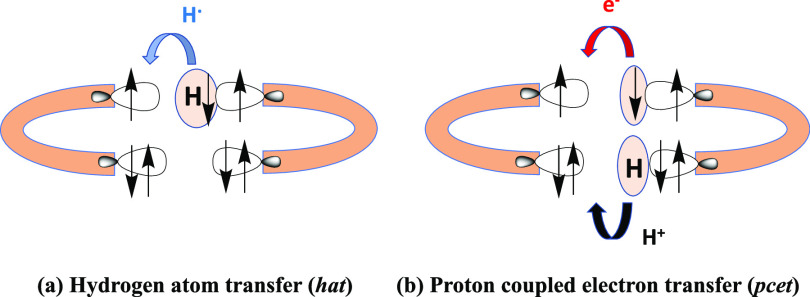
Picture Showing the Main Features of the Hydrogen Atom Transfer and Proton-Coupled Electron Transfer Mechanisms

Indeed, eq 1 shows that the radical R^•^ abstracts one hydrogen atom generating a new radical A^•^, which can react with other species initiating a chain of reactions that occur in most oxidative processes, either in biological systems or in atmospheric and environmental chemistry.^2−9^ The most common knowledge of the mechanism for the hydrogen abstraction is that radical R^•^ approaches the H–A bond, producing the homolytic breaking of the covalent H–A bond and the making of a covalent R–H bond. It corresponds to a hydrogen atom transfer process (*hat*), as shown in Scheme 1a, and it is well established that its energy barrier depends on the bond dissociation energy (BDE) of the H–A bond.^3,10−12^

However, there is a different kind of process occurring in eq 1 as displayed in Scheme 1b. This mechanism implies the simultaneous transfer of one electron from the H–A reactant (from a lone pair not involving the H–A bond) and the H(−A) proton to R^•^ and corresponds to a proton-coupled electron transfer (*pcet*). When this process is possible, there is no need for the breaking of the H–A covalent bond, avoiding its energetic cost and generating a more favorable reaction path.^3,13−16^

In the context of the chemistry of the atmosphere, the abstraction of hydrogen atoms by radicals is among the most important oxidation reactions, and most of them follow a *hat* mechanism. However, the reaction of the OH^•^, Cl^•^, ClO^•^, NH_2_^•^ radicals with atmospheric acids follows a *pcet* mechanism.^17−27^ Very recently, it has been also reported that the triplet electronic state of SO_2_ can abstract one hydrogen atom from alkanes, via the *hat* mechanism,^28^ and one hydrogen atom from water^29^ via the *pcet* mechanism.^30,31^ These results suggest the importance of the chemistry of the excited states of SO_2_.

Sulfur dioxide is an important pollutant which is produced by anthropogenic fossil fuel burning^32^ or emitted from natural sources such as volcano’s eruption^33^ and oxidation of dimethyl sulfide.^34^ Its chemistry is associated with the tropospheric formation of sulfuric acid, a constituent of acid rain and sulfate aerosols.^35−37^ The triplet excited states of sulfur dioxide are accessed by near-UV solar radiation (with absorption band from 240 to 330 nm) and have a lifetime of 7.97 ± 1.7 × 10^–4^ s,^38^ indicating that they are able to react with atmospheric trace gases. Indeed, the excited triplet states of sulfur dioxide have been shown to react with different species,^39−46^ and the reaction of ^3^SO_2_ with H_2_O at the surface of water droplets constitutes a source of atmospheric sulfuric acid.^31,47−49^

In order to further explore the reactivity of the triplet excited state of SO_2_ in the gas phase, in this work, we have investigated its reactions with formic and nitric acids according to eqs 2 – 4.
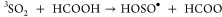
2
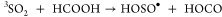
3
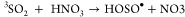
4

Formic and nitric acids display very different gas phase acidities (see below), but both are important atmospheric trace gases, and we aim to analyze the possible impact of the foregoing reactions on the chemistry of the Earth’s atmosphere.

## Methods

All the reactions have been investigated with the B3LYP^50^ density functional using the aug-cc-pVTZ basis set.^51^ It is worth noting that the B3LYP^50^ density functional is suitable to describe in particular the NO_3_ radical (formed in the reaction with HNO_3_), which presents a doublet instability when using various other functionals.^24^ In the case of the reaction of SO_2_(^3^B_1_) with HCOOH, however, one transition state could not be located with the B3LYP functional, despite many trials. Hence, we decided to carry out for some selected stationary points of this reaction some additional calculations using the BH&HLYP^52^ and M0–62X^53^ density functionals, along with the aug-cc-pVTZ basis set.^51^ At these levels of treatment, we have performed harmonic frequency calculations to verify the nature of the stationary points and to obtain zero point, thermal, and Gibbs free energies. In addition, we have carried out IRC^54^ calculations to confirm the connectivity of the transition states. Moreover, some structures were also optimized employing the coupled cluster method with single and double excitations, and a perturbative treatment of all connected triplet excitations (CCSD(T)),^55^ employing the 6-311+G(2df,2p) basis set.^56,57^ The bonding characteristics of some selected stationary points have been examined according to the topological theory of atoms in molecules (AIM).^58^

Finally, in order to obtain accurate relative energies, we have performed single-point energy calculations at all optimized geometries with the CCSD(T) method employing the aug-cc-pVTZ, aug-cc-pVQZ, and complete CBS basis sets.^51,56,57,59,60^ In all these calculations, we have looked at the T1 diagnostic^61^ with regard to the possible multireference character of the CCSD wave function.

The rate constants have been calculated by numerical integration considering a set of elementary processes for which Rise–Ramsperger–Kassel–Marcus (RRKM)^62^ calculations have been carried out. The zero-curvature tunneling effects for an unsymmetrical Eckart barrier^63^ have been considered, and the effect of the pressure has been also taken into account. For all of these calculations, the CCSD(T)/CBS relative energies have been considered as well.

The quantum chemical calculations carried out in this work were performed by using Gaussian^64^ and ORCA^65^ program packages, the kinetical calculations were performed with Multiwell^66^ program, and the numerical integration has been done with a house made python program.^67^ Further details of these results are reported in theSupporting Information.

## Results and Discussion

As usual in many gas phase reactions, each elementary reaction begins with the formation of a prereactive complex that precedes the transition state and forms a postreactive complex, before the release of the products. Along this text, the different stationary points of the reaction of ^3^SO_2_ with HCOOH are labeled by the letter A, followed by the acronym CR for prereactive complexes, TS for transition states, or CP for postreactive complexes, and by a number. In a similar way, the different stationary points for the reaction of ^3^SO_2_ with HNO_3_ are labeled by the letter B followed by CR, TS, or CP, and a number. For the sake of comparison, we have also updated our investigation on the ^3^SO_2_ + H_2_O reaction.^30^

## The Triplet States of SO_2_

The ground and low-lying electronic states of SO_2_ are very well-studied experimentally^68−70^ and theoretically,^71−73^ and our results, previously reported in thesupporting informationof ref^30^, are in a very good agreement with the data from the literature. For the sake of completeness, we have collected in Table 1 the electronic characterization, optimized geometrical parameters, and adiabatic excitation energies of the ground and low-lying electronic triplet states of SO_2_, which are relevant for the present work.

**Table 1:** Adiabatic Excitation Energies (in eV, Including ZPE), and Optimized Geometrical Parameters for the Ground Electronic State and Low-Lying Triplet States of SO_2_^a^^b^

state	electronic characterization	r(SO)	a(OSO)	ΔE (eV)
X^1^A_1_	.···8a_1_^2^2b_1_^2^5b_2_^2^1a_2_^2^	1.451	118.2	0.00
a^3^B_1_	···.7a_1_^2^2b_1_^2^5b_2_^2^1a_2_^2^8a_1_^1^3b_1_^1^	1.515	124.8	3.15
b^3^A_2_	.···.8a_1_^2^2b_1_^2^4b_2_^2^1a_2_^2^3b_1_^1^5b_2_^1^	1.550	93.6	3.19
c^3^B_2_	.···.8a_1_^2^2b_1_^2^5b_2_^2^1a_2_^1^3b_1_^1^	1.576	104.7	3.39

a Distances are in angstroms and angles in degrees.

b Geometries optimized at B3LYP/aug-cc-pVTZ and relative energies computed at CCSD(T)/CBS//B3LYP/aug-cc-pVTZ. This table has been reproduced with permission from the supplementary information of ref (30). Copyright 2019 Royal Society of Chemistry.

The triplet excited states of sulfur dioxide can be accessed in the UV region from the X^1^A_1_ ground state either through direct spin-forbidden excitation or through excitation to the A^1^B_1_ excited state, followed by intersystem crossing to the triplet states. Moreover, the a^3^B_1_, b^3^A_2_, and c^3^B_2_ triplet electronic states lie very close to each other in energy and their potential energy surfaces cross,^72−75^ so that they should be populated and considered as reactants.

Regarding the reactivity of these species with formic and nitric acids, an exhaustive search has shown that all elementary reactions take place in the plane of the SO_2_ moiety and involve the interaction of the acids with either the 8a_1_ or the 5b_2_ σ single occupied orbitals of the triplet states, but not with the out of plane π orbitals 3b_1_ or 1a_2_. Therefore, only the a^3^B_1_ or b^3^A_2_ electronic states of sulfur dioxide can be involved in the reactions, but not the c^3^B_2_ electronic state.

## The Reaction of the Triplet Excited States of SO_2_ with HCOOH

**Figure 1:**
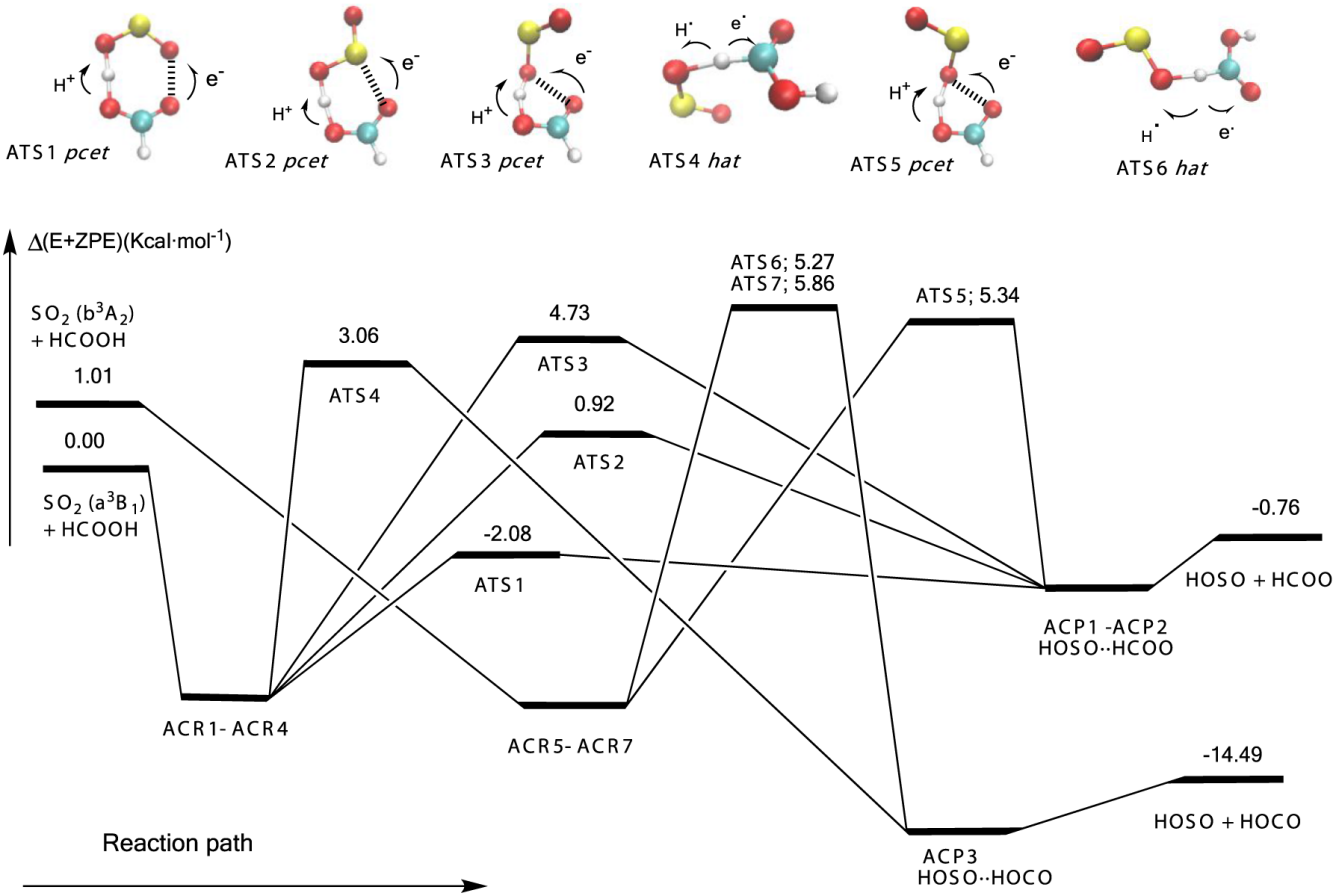
Schematic potential energy surface for the ^3^SO_2_ + HCOOH. The values correspond to CCSDT/CBS//B3LYP/aug-cc-pVTZ) calculations, except for ATS4, which corresponds to CCSDT/CBS//BH&HLYP/aug-cc-pVTZ) calculations. We have omitted the relative energies of the prereactive and postreactive complexes for the sake of clarity, which are collected in the Supporting Information. Energies (kcal mol^–1^) include zero-point energies.

**Figure 2:**
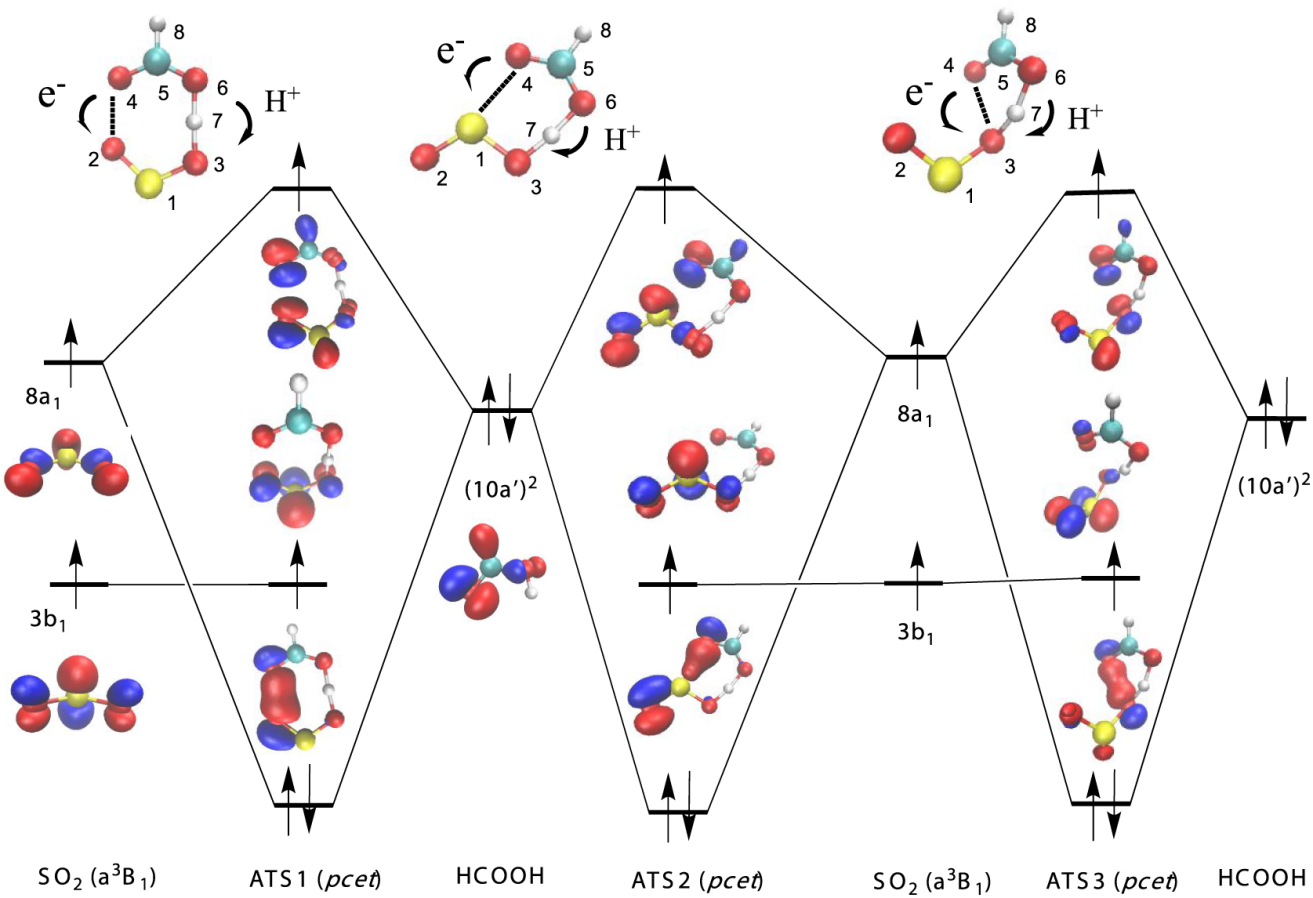
Orbital diagram for the *pcet* mechanisms of the SO_2_ (a^3^B_1_) + HCOOH reaction.

**Figure 3:**
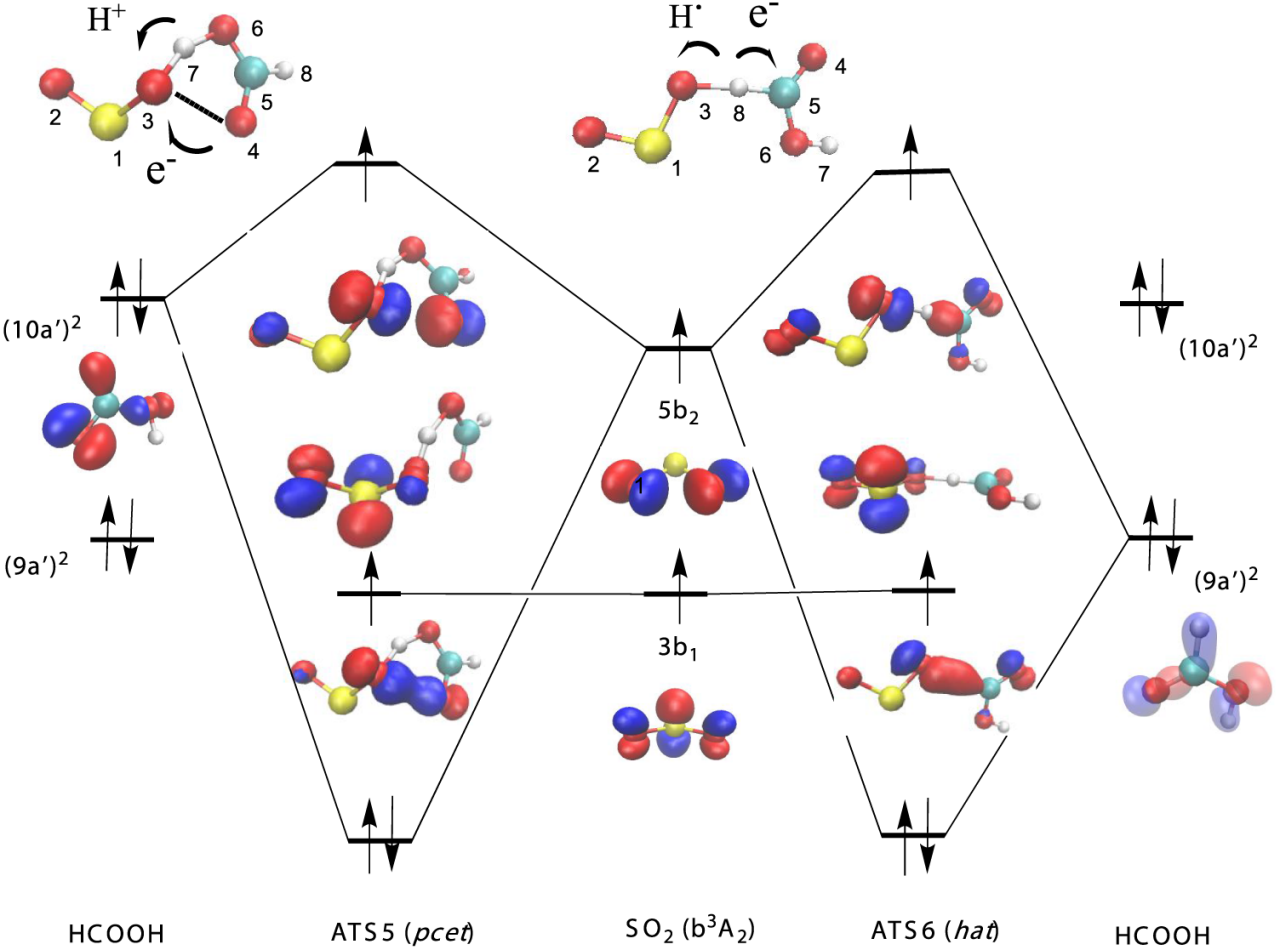
Orbital diagram for the *pcet* and *hat* mechanisms for the SO_2_ (a^3^A_2_) + HCOOH reaction.

Figure 1 shows a schematic potential energy surface of the reaction of the triplet excited states of sulfur dioxide with formic acid, and Figures 2 and 3 contain the most relevant electronic features at the transition states. Table S1 and Figure S1 contain the relative energies, enthalpies, and free energies computed with the different theoretical approaches and a diagram showing all connections of the stationary points.

We have found four elementary reactions for the reaction of a^3^B_1_ + HCOOH (via ATS1, ATS2, ATS3, and ATS4) and three elementary reactions for the reaction of b^3^A_2_ + HCOOH (via ATS5, ATS6, and ATS7). The reaction paths through ATS1, ATS2, ATS3, and ATS4 correspond to reaction 2 (abstraction of the acidic hydrogen), and the reaction paths through ATS4, ATS6, and ATS7 correspond to reaction 3 (abstraction of the formyl hydrogen).

The electronic features of ATS1, ATS2, and ATS3 are displayed in Figure 2 and allow us to classify these elementary reactions as having a *pcet* reaction mechanism. These three processes abstract the acidic hydrogen of the acid and involve the interaction of the unpaired electron over the 8a_1_ orbital of ^3^SO_2_ (a^3^B_1_, (···3b_1_^1^8a_1_^1^)) with the lone pair (10a’)^2^ of HCOOH in a two center-three electron system. The electronic density of the 10a’ orbital of formic acid lies mainly over the oxygen atom of the carbonyl group, and the electronic distribution of the 8a_1_ orbital of ^3^SO_2_ is delocalized over the three atoms, lying in the molecular plane (see Figure 2). Therefore, there are three different possibilities for the topological interactions of the ^3^B_1_ electronic state of SO_2_ with the acid, leading to three different reaction paths with ATS1, ATS2, and ATS3 transition states.

In ATS1, the electron is transferred from the oxygen atom of the carbonyl group (O4, see Figure 2 for atom numbering) to one oxygen atom of ^3^SO_2_ (O2), while the acidic proton jumps to the other oxygen atom (O3) of sulfur dioxide in a seven-membered ring structure.

In ATS2, the electron transferred goes to the sulfur atom (from O4 to S1), and the proton moves to one of the oxygen atoms of ^3^SO_2_ (O2) in a six-member ring structure. It is interesting to mention that sulfur atom acts as an electron acceptor in this case, though it can also act as an electron donor in *pcet* driven processes, as reported for the reaction of hydropersulfides with hydroxyl radical.^17^

ATS3 has a five-membered ring structure where the electron is transferred from O4 of the acid moiety to O3 of the SO_2_ moiety, whereas the acidic proton jumps to the same oxygen atom (O3) of sulfur dioxide. It is interesting to note that ATS3 has the same scaffold as other transition states with a *pcet* mechanism, as in the reaction of ^3^SO_2_ with H_2_O,^30^ or in the reaction of the OH, NH_2_, Cl, ClO radicals with several acids.^17−27^

In summary, all three *pcet* transition states can be described by a two center-three electron system, whereas the single occupied 3b_1_ orbital of the triplet does not participate in the reaction and acts as a spectator. Actually, the AIM analysis of the electron charge density displayed in Table 2 indicates the existence of a bond critical point between the two atoms involved in the electron transfer, with a small electron charge density value (ρ(r_b_)) and a positive value of the Laplacian (∇^2^ρ(r_b_)). This result is characteristic of a noncovalent interaction, as described previously in the literature for a *pcet* mechanism.^13^

**Table 2:** Electron Density (ρ(r_b_) in e·bohr^–3^), Laplacian of the Electron Density (∇^2^ρ(r_b_) in e·bohr^–5^), and Local Energy Density (E(r_b_) in hartree·bohr^–3^) for the Bond Critical Points (r_b_) at ATS1, ATS2, and ATS3

transition state	bond^a^	ρ (r_b_)	∇^2^ρ (r_b_)	E (r_b_)
ATS1	O4–O2	0.039943	0.161482	0.004015
ATS2	O4–S1	0.042546	0.105987	–0.001743
ATS3	O4–O3	0.032215	0.124156	0.003767

a See Figure 2 for atom numbering.

On the other side, the reaction via ATS4 involves the abstraction of the formyl hydrogen by sulfur dioxide (reaction 3) and follows a conventional hydrogen atom transfer mechanism (see below).

The electronic features of ATS5 and ATS6 are plotted in Figure 3 and correspond to the interaction of the orbital 5b_2_ of the excited stated b^3^A_2_ (···3b_1_^1^5b_2_^1^) of ^3^SO_2_ with the lone pair (10a’)^2^ of HCOOH. The elementary reaction through ATS5 follows a *pcet* mechanism and has the same features than ATS3 describe above, namely it has a five-membered ring structure where the electron is transferred from O4 of the acid moiety to O3 of the SO_2_ moiety, and the acidic proton jumps to the same oxygen atom (O3) of sulfur dioxide. Both transition states differ in the S–O–S angle of the sulfur dioxide moiety, according to the difference in the corresponding angle between the a^3^B_1_ and b^3^A_2_ electronic states of the ^3^SO_2_ reactant (see Figure S1). Figure 3 also shows that the elementary reaction through ATS6 proceeds by the interaction of the (9a’)^2^ of formic acid with the single occupied 5b_2_ orbital of sulfur dioxide, in a three center-three electron system, which corresponds to a concerted breaking of the C–H bond and forming of the H–O(SO) bond, with a *hat* mechanism (reaction 3). Please note that the elementary reactions via ATS4 and ATS7 have the same *hat* mechanism, and again, the single occupied 3b_1_ orbital of the triplet does not participate in the reaction and acts as a spectator.

Figure 1 and Table S1 show that the reaction of ^3^SO_2_ with HCOOH leading to the formation of HOSO + HCOO (reaction 2) is computed to be exothermic by 0.76 kcal·mol^–1^, whereas the formation of HOSO + HOCO, (reaction 3) is computed to be exothermic by 14.49 kcal·mol^–1^. The reaction path involving the seven-membered-ring ATS1 has the lowest energy barrier, and the transition state lying below the energy of the separate reactants (−2.08 kcal·mol^–1^). The energy barrier of six-membered-ring ATS2 is computed to be 0.92 kcal·mol^–1^. In ATS1, the electron is transferred to the terminal oxygen atom of ^3^SO_2_, which is more electronegative than the sulfur atom, as in ATS2, making the transfer of the electron easier and therefore resulting in a smaller energy barrier. Besides, the reaction paths via ATS3 and ATS5 with a five-membered ring structure have a higher energy barrier (about 4.3 kcal·mol^–1^) due to the more constrained geometry at the transition states. For the hydrogen atom transfer processes ATS4, ATS6, and ATS7 (reaction 3), we have calculated energy barriers of 3.06 kcal·mol^–1^ relative to the reactants SO_2_(^3^B_1_) + HCOOH, and 4.26 and 4.86 kcal·mol^–1^ respectively relative to the reactants SO_2_(^3^A_2_) + HCOOH. It is worth mentioning here that, despite an exhaustive search, we could not find a reaction path involving the abstraction of the acidic hydrogen by the triplet with a *hat* mechanism, and all efforts converged to a *pcet* process.

It is also interesting to point out that our results predict the abstraction of the acidic hydrogen by the triplet state of sulfur dioxide rather than the abstraction of the formyl hydrogen, despite the fact that the bond dissociation energy (BDE) of the C–H bond (96.2 ± 0.7 kcal·mol^–1^) involved in *hat* mechanisms is smaller than the BDE of the O–H bond (112.2 ± 3.1 kcal·mol^–1^).^76^ This can be explained by the fact that the abstraction of the acidic hydrogen takes place via *pcet* mechanisms (ATS1 and ATS2) in a similar manner as discussed in the oxidation of formic acid by hydroxyl radical.^18,77^

## The Reaction of the Triplet Excited States of SO_2_ with HNO_3_

**Figure 4:**
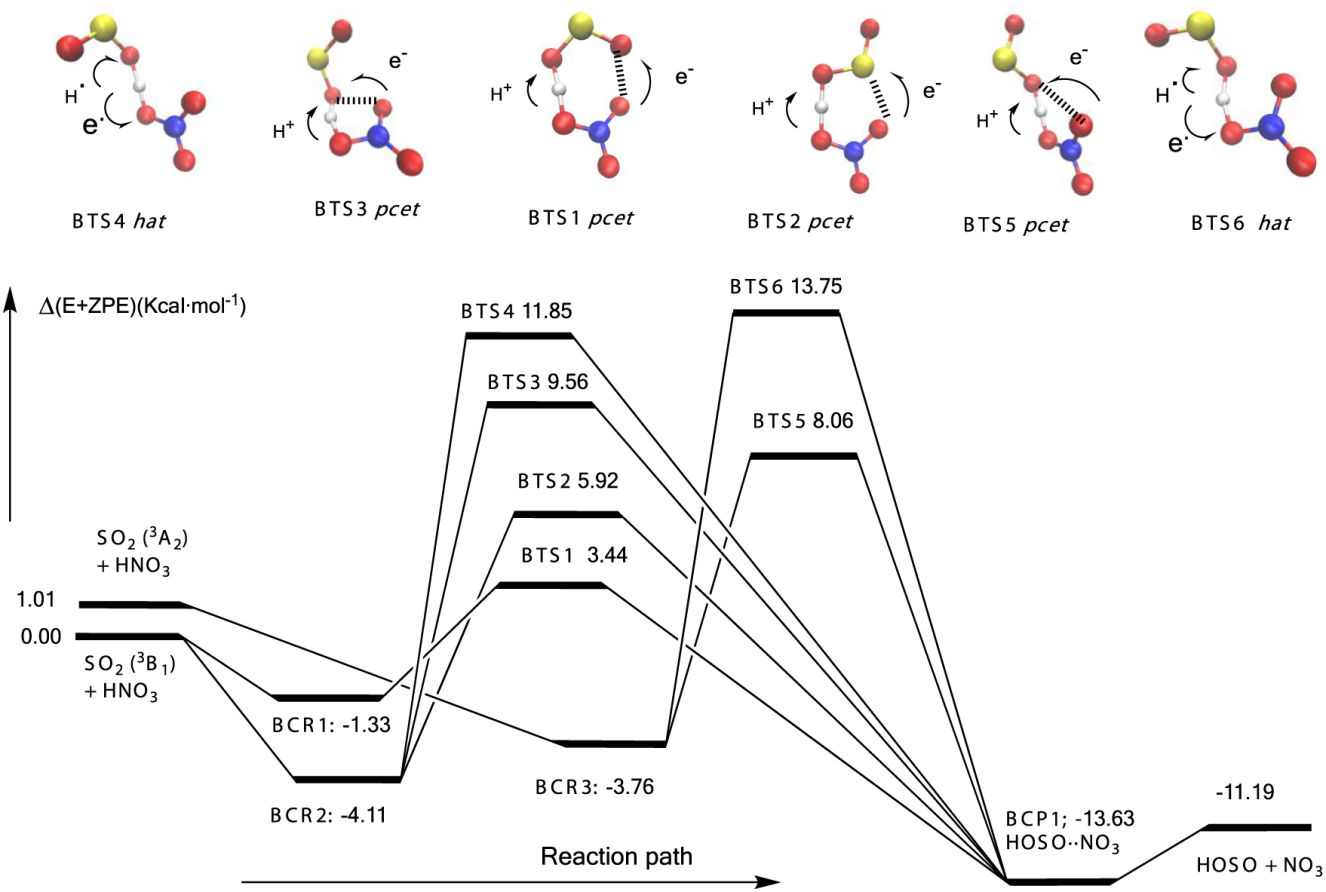
Schematic potential energy surface for ^3^SO_2_ + HNO_3_. The values correspond to CCSDT/CBS//B3LYP/aug-cc-pVTZ). Energies (kcal·mol^–1^) include zero-point energies.

The reaction of the triplet states of sulfur dioxide with nitric acid follows the same patterns as discussed above for the reaction with formic acid. Figure 4 shows that there are four elementary reactions for the reaction of SO_2_ (a^3^B_1_) with HNO_3_ (via BTS1 – BTS4) and two elementary reactions for the reaction of SO_2_ (b^3^A_2_) with HNO_3_ (via BTS5 and BTS6). All elementary reactions lead to the formation of HONO + NO_3_ radicals, reaction 3, which is computed to be exothermic by 11.19 kcal·mol^–1^ (see Figure 4 and Table S2).

The geometrical and electronic features of BTS1, BTS2, BTS3, and BTS5 are the same as the corresponding transition states of the reaction with formic acid (ATS1, ATS2, ATS3, and ATS5) described above and follow a *pcet* mechanism, whereas the elementary reactions through BTS4 and BTS6 involve abstraction of the acidic hydrogen via a *hat* mechanism.

Figure 4 and Table S2 show that the calculated energy barriers range between 3.5 and 9.5 kcal·mol^–1^ for the elementary reactions with a *pcet* mechanism and between 12 and 14 kcal·mol^–1^ for the *hat* processes. Again, the energy barrier for the *pcet*-driven reactions is much smaller than the *hat*-driven reaction as discussed above and elsewhere^13^ and in line with the oxidation of nitric acid with hydroxyl radical.^21^

**Figure 5:**
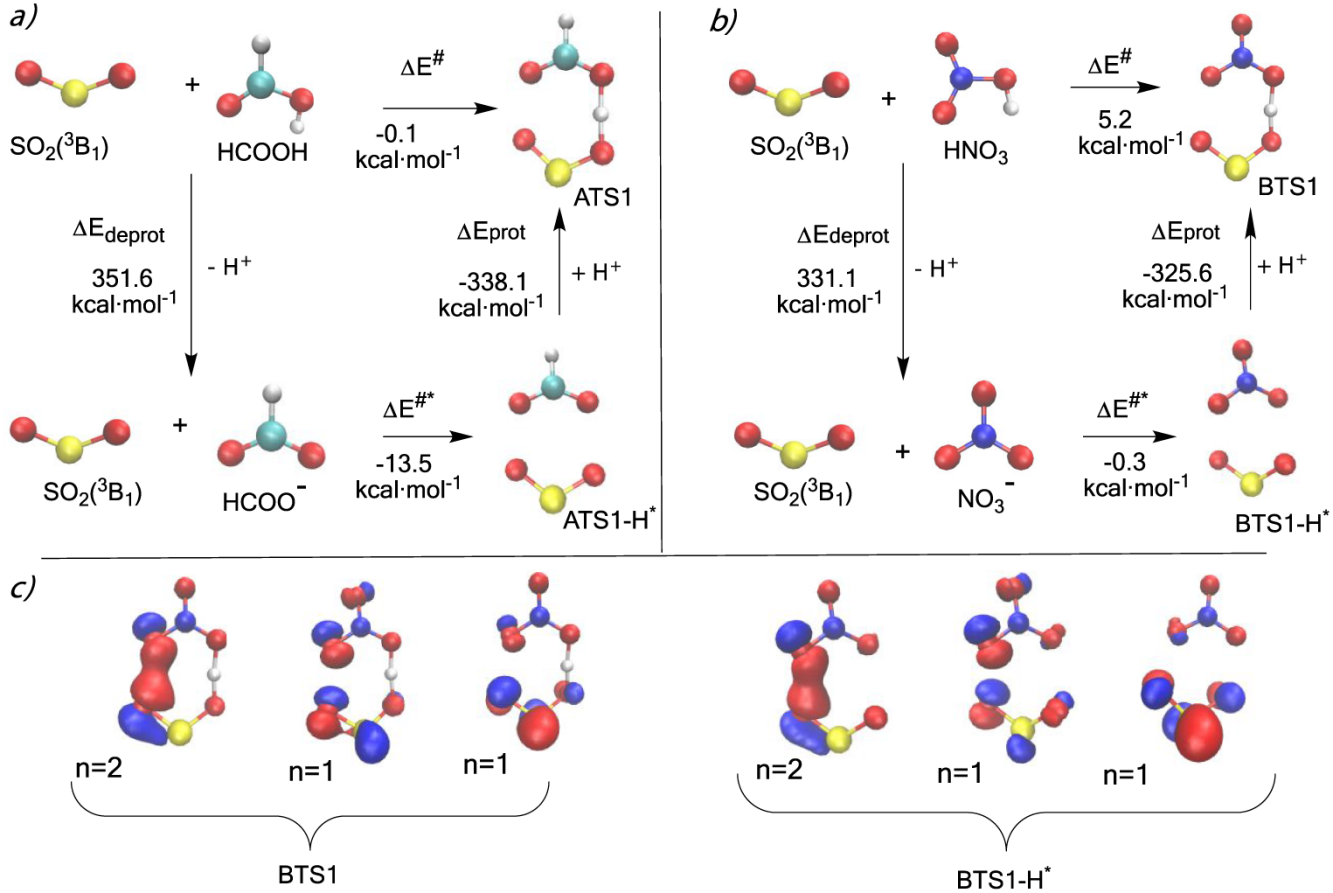
Thermodynamic cycles comparing the deprotonation energy of the reactants (SO_2_(^3^B_1_) with HCOOH and HNO_3_) with those of the transition states ATS1 and BTS1, along with the energetic requirements for the formation of these structures (Figure 5a,5b respectively). Figure 5c shows the electronic features of the most relevant natural orbitals of BTS1 and BTS1-H*.

It is also interesting to point out that the lowest energy barriers for the oxidation of formic and nitric acids by the triplet states of sulfur dioxide are characterized by *pcet* processes with the same electronic and geometric features at the transition states. However, our results show that the energy barriers in the case of the oxidation of nitric acid are about 5 kcal·mol^–1^ greater than the corresponding energy barrier for the reaction with formic acid. In order to understand these differences in the energy barriers in both systems, we have considered a thermodynamic cycle as proposed by Mayer and coworkers,^78^ shown in Figure 5a,b. The first step corresponds to the deprotonation energy of the reactants (formic or nitric acid); the second step corresponds to evaluate the energy of formation of the transition state structure without the proton being transferred (ATS1-H* and BTS1-H*); and the third step consists of calculating the energy liberated while bringing the proton to these structures to form the transition states ATS1 and BTS1, respectively. Please note that ATS1-H* and BTS1-H* are not stationary points, and therefore, we evaluate just energies. We have noticed that the structures without the proton being transferred have the same electronic distribution than the corresponding transition states, as it is shown in Figure 5c for BTS1 and BTS1-H*, where we have plotted the electronic features of the most relevant natural orbitals.

Figure 5a,b show that the formation of HCO_2_^–^ requires 20.5 kcal·mol^–1^ more than the formation of NO_3_^–^, but the energy liberated in forming ATS1 from ATS1-H* is 12.6 kcal·mol^–1^ greater than the energy liberated in forming BTS1 from BTS1-H*. Moreover, bringing the triplet SO_2_ and the HCOO anion to the ATS1-H* geometry liberates 13.2 kcal·mol^–1^ more energy than bringing the triplet SO_2_ and NO_3_ anion to BTS1-H* geometry, so that the formation of ATS1 is 5.3 kcal·mol^–1^ more favorable than the formation of BTS1. In connection with these results, it is worth mentioning that our computed Gibbs free energies for the deprotonation of formic and nitric acid (343.5 and 324.5 kcal·mol^–1^) compare very well with the experimental values (344.67 ± 0.62 and 325.5 ± 0.2, respectively).^79,80^

## Relevance of the Results and Atmospheric Implications

The broadest and most important conclusion to be drawn from the reactions investigated in this work is that the reactivity of ^3^SO_2_ is very versatile with respect to atmospheric acids. The electronic and topological features of the 8a_1_ molecular orbital of the ^3^B_1_ electronic state allow for multiple interaction sites leading to three different reaction paths, and all of them have a proton-coupled electron transfer nature. The fact that the 3b_1_ orbital does not participate at all in the reactions indicates that the triplet electronic state of sulfur dioxide acts as a pure radical species. Moreover, our analysis shows that the greater affinity of the ^3^SO_2_ with the HCOO^–^ anion is responsible for the lowest *pce*t barrier for the reaction of the triplet sulfur dioxide with formic acid.

Beyond the fundamental interest in the mechanistic peculiarities of the processes discussed in the previous section, the reactions investigated in this work have been found to be highly relevant in the chemistry of the atmosphere. In Table 3, we have collected the calculated rate constants for the reaction of ^3^SO_2_ with HCOOH, HNO_3_, and water vapor at different altitudes in the Earth’s atmosphere. At ground level, the rate constants for the reaction of ^3^SO_2_ with HCOOH and HNO_3_ are computed to be 3.54 × 10^–13^ and 3.97 × 10^–17^ cm^3^·molecule^–1^·s^–1^, respectively. The rate constant for the reaction with formic acid is about 4 orders of magnitude greater than the reaction with water, but the rate constant of the reaction with nitric acid is almost two times smaller than the reaction with water vapor. Table 3 also shows that at different altitudes in the Earth’s atmosphere the rate constants for the reaction with formic acid can reach up to 6 orders of magnitude greater than the reaction with water, but the rate constant of the reaction with nitric acid is 2 orders of magnitude greater than that of the reaction with water just at very high altitude in the Earth’s atmosphere (49 km). Full details of these calculations are described in the Supporting Information.

**Table 3:** Rate Constants, in cm^3^·molecule^–1^·s^–1^, for the Reactions of ^3^SO_2_ with HCOOH (*k*_1_), with HNO_3_ (*k*_2_), and with H_2_O (*k*_3_), at Different Altitudes (in km) in the Earth’s Atmosphere, along with the Concentration of Water Vapor (in molecules·cm^–3^)

altitude^a^	*T*^a^	*P*^a^	*k*_1_ (^3^SO_2_ + HCOOH)	*k*_2_ (^3^SO_2_ + HNO_3_)	*k*_3_ (^3^SO_2_ + H_2_O)	[H_2_O]^b^	*k*_*3*_[H_2_O]/*k*_1_^c^	*k*_3_ [H_2_O]/*k*_2_^d^
0	298.1	1.00	3.54 × 10^–13^	3.97 × 10^–17^	6.35 × 10^–17^	7.37 × 10^17^	1.32 × 10^14^	1.18 × 10^18^
5	259.3	0.535	5.68 × 10^–13^	1.72 × 10^–17^	2.29 × 10^–17^	2.41 × 10^16^	9.72 × 10^11^	3.21 × 10^16^
10	229.7	0.266	9.37 × 10^–13^	8.54 × 10^–18^	9.35 × 10^–18^	4.92 × 10^15^	4.91 × 10^10^	5.39 × 10^15^
15	212.6	0.12	1.36 × 10^–12^	5.70 × 10^–18^	2.36 × 10^–18^	1.96 × 10^13^	3.40 × 10^7^	8.12 × 10^12^
20	215.5	0.054	1.27 × 10^–12^	6.11 × 10^–18^	5.05 × 10^–18^	9.56 × 10^12^	3.80 × 10^7^	7.90 × 10^12^
25	218.6	0.025	1.18 × 10^–12^	6.57 × 10^–18^	1.97 × 10^–18^	5.21 × 10^12^	8.70 × 10^6^	1.56 × 10^12^
30	223.7	0.011	1.06 × 10^–12^	7.41 × 10^–18^	9.94 × 10^–19^	2.62 × 10^12^	2.46 × 10^6^	3.51 × 10^11^
35	235.1	0.005	8.46 × 10^–13^	9.73 × 10^–18^	4.20 × 10^–18^	1.31 × 10^12^	6.52 × 10^6^	5.65 × 10^11^
40	249.9	0.003	6.56 × 10^–13^	1.38 × 10^–17^	3.16 × 10^–19^	6.44 × 10^12^	3.10 × 10^6^	1.47 × 10^11^

a Altitude in km, *T* in K, and *P* in atm.

b Taken from ref^81^.

c Minimum concentration of HCOOH in which ^3^SO_2_ + HCOOH will compete with ^3^SO_2_ + H_2_O.

d Minimum concentration of HNO_3_ in which ^3^SO_2_ + HNO_3_ will compete with ^3^SO_2_ + H_2_O.

Taking into account these kinetic constants, the concentration of water vapor at different altitudes in the atmosphere, and eqs 4 – 6, we have estimated the concentrations of HCOOH and HNO_3_ at which these reactions become significant.
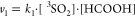
5
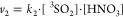
6
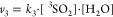
7

Table 3 shows that for these reactions to be competitive, it would be required HCOOH concentrations of about 1 × 10^14^ molecules·cm^–3^ at ground level, 3 × 10^7^ molecules·cm^–3^ at 15 km high, and 3 × 10^6^ molecules·cm^–3^ at 40 km high, and concentrations of HNO_3_ about 1 order of magnitude greater. Measures of atmospheric HCOOH concentrations range between 2 × 10^9^ and 2 × 10^10^ molecules·cm^–3^ in average^82,83^ and up to 1.35 × 10^13^ molecules·cm^–3^ at 4.5 km high^84^ and 8.9 × 10^8^ molecules·cm^–3^ at 15 km high,^85^ Concentrations of HNO_3_ have been measured to range between 1.16 × 10^10^ and 1.32 × 10^12^ molecules·cm^–3^,^86,87^ with values up to 2.0 × 10^10^ molecules·cm^–3^ in the upper troposphere and in the stratosphere.^88^

All these values suggest that the reactions of triplet sulfur dioxide with formic acid can compete with the reaction with water at the upper troposphere and in the stratosphere and therefore play a role in the chemistry of the atmosphere. In deep contrast, the reaction with nitric acid cannot compete with that with water vapor. Our results, along with eqs S3–S6 of ref^47^, which allow taking into account the possible role of deactivation processes from the singlet and triplet excited states of SO_2_ to the ground state, have been combined to provide an estimation of the reaction rate for the formation of HCOO in the reaction of ^3^SO_2_ + HCOOH compared with the formation of OH radicals in the reaction of ^3^SO_2_ + H_2_O. For instance, taking the values displayed in Table 3, and concentrations of X^1^SO_2_ and HCOOH of 1.0 × 10^10^ molecules·cm^–3^, and 2.0 × 10^9^ molecules·cm^–3^, respectively we estimate reaction rates for the formation of HCOO (reaction of ^3^SO_2_ + HCOOH) of 3.98 × 10^3^, 1.38 × 10^4^, and 1.17 × 10^6^ molecules·cm^–3^·s^–1^ at 15, 20, and 30 km of altitude in the Earth’s atmosphere, compared with the reaction rates of 1.89 × 10^4^, 3.73 × 10^3^, and 1.44 × 10^2^ molecules·cm^–3^·s^–1^ at the same altitudes for the formation of OH radicals in the reaction of ^3^SO_2_ + H_2_O. Please note that HCOO can easily decompose into H and CO_2_,^89^ that is also of interest in the chemistry of the atmosphere.

The wider implication of our study is that the triplet state chemistry in the atmosphere is possibly more significant than is commonly thought, and the present calculations show just how much more reactive ^3^SO_2_ is in atmospheric reactions involving hydrogen atom processes. The findings of this work open the door to possibilities and significance of hydrogen abstraction reactions involving triplet state species in the atmosphere, for which few such studies are known.

## Supporting Information Available

Further details on the theoretical methods, reaction mechanisms, electronic features, relative energies, kinetic study, the reaction of ^3^SO_2_ with H_2_O, geometrical parameters, and Cartesian coordinates are provided (PDF)
