# Supplementary material for: Triplet State Radical Chemistry: Significance of the Reaction of 3SO2 with HCOOH and HNO3
Source: J Am Chem Soc. 2024 May 9;146(20):14297–306. doi: 10.1021/jacs.4c03938 (PMC11117184; doi:10.1021/jacs.4c03938)
Supplement: Supplementary file 1 — ja4c03938_si_001.pdf [file ja4c03938_si_001.pdf]

Triplet State Radical Chemistry: Significance of the Reaction of $^3SO_2$ with HCOOH and HNO_3

Josep M. Anglada, [*a] Marilia T. C. Martins-Costa,[b] Joseph S. Francisco,[c] and Manuel F. Ruiz-López[b]

a) Departament de Química Biològica (IQAC – CSIC), c/ Jordi Girona 18, E-08034 Barcelona, Spain
b) Laboratoire de Physique et Chimie Théoriques, UMR CNRS 7019, University of Lorraine, CNRS, BP 70239, 54506 Vandoeuvre-lès-Nancy, France
c) Department of Earth and Environmental Science and Department of Chemistry, University of Pennsylvania, Philadelphia, PA, USA 19104-6316

(Supporting Information)

The reaction of $^3B_1$ and $^3A_2$ electronic states of SO_2 with HCOOH, HNO_3 and H_2O.
Along the main text, the different stationary points of the reaction of the $^3B_1$ and $^3A_2$ electronic states of $^3SO_2$ with HCOOH, HNO_3, and H_2O are labelled by the letters A, B, and C respectively, followed by the acronym CR for the pre-reactive complexes, TS for the transition states and CP for the post-reactive complexes, and followed by a number. In a previous article, we have investigated the reaction of $^3SO_2$ with H_2O,[1] and in this work we have updated the results of that study with the aim of getting rate constants for all these reactions at the same theoretical level.

The reaction of the $^3SO_2$ excited states with HCOOH.
Figure S1 shows how the different stationary points (reactants, pre-reactive complexes, transition states, post-reactive complexes, and products) are connected. The schematic potential energy surface is displayed in Figure 1 of the main text.

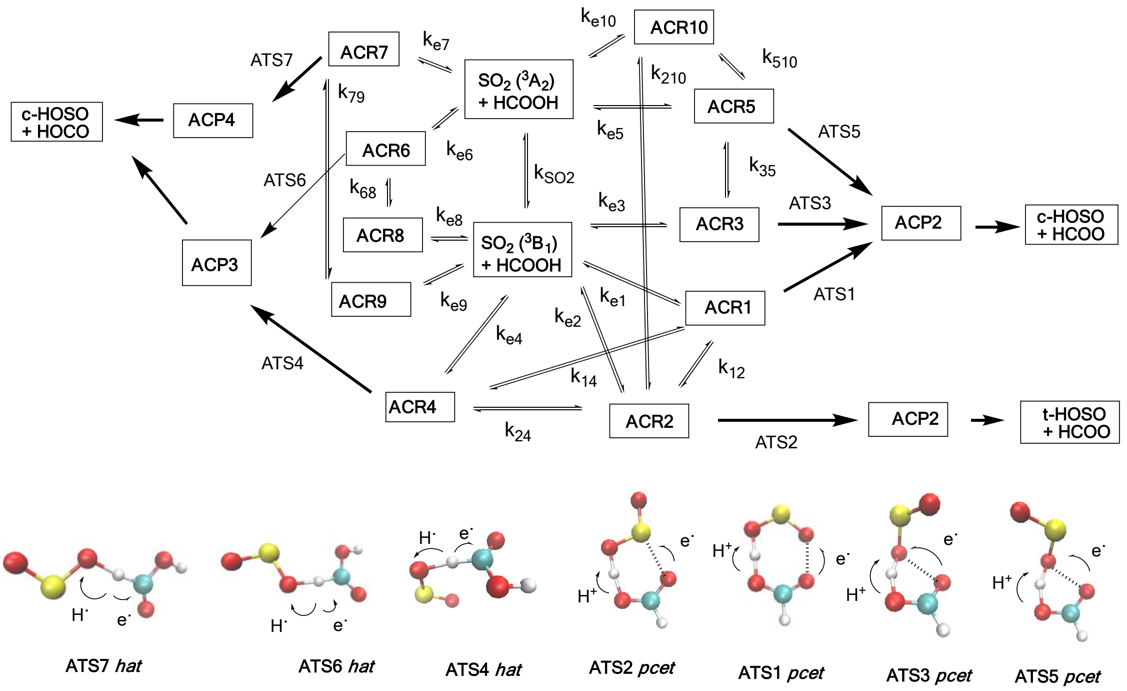

Figure S1: Scheme for the connection of the stationary points for the reaction between the electronic states $^3B_1$ and $^3A_2$ of SO_2 with HCOOH.

As pointed out in the main text, we have employed the B3LYP/aug-cc-pvtz approach to optimize and characterize all stationary points, although some stationary points of interest were further optimized using the BH&HLYP/aug-cc-pVTZ, M06-2X/aug-cc-pVTZ, and CCSD(T)/6-311+G(2df,2p) theoretical approaches. Thus, ACR1, ACR2, ACR3, ACR3, ATS1, ATS2, ATS3, and ATS4 were optimized with all mentioned theoretical approaches, although we failed to find ACR4 and ATS4 at B3LYP level of theory. The corresponding Cartesian coordinates are listed below in Tables S6 to S9. Figure S2 shows the most relevant geometrical parameters of all stationary points. The geometrical parameters optimized with the different theoretical approaches differ in less than 0.03 Angstroms, except for some hydrogen bond distances in the case of pre-reactive complexes, that differ in up to 0.25 Angstroms.

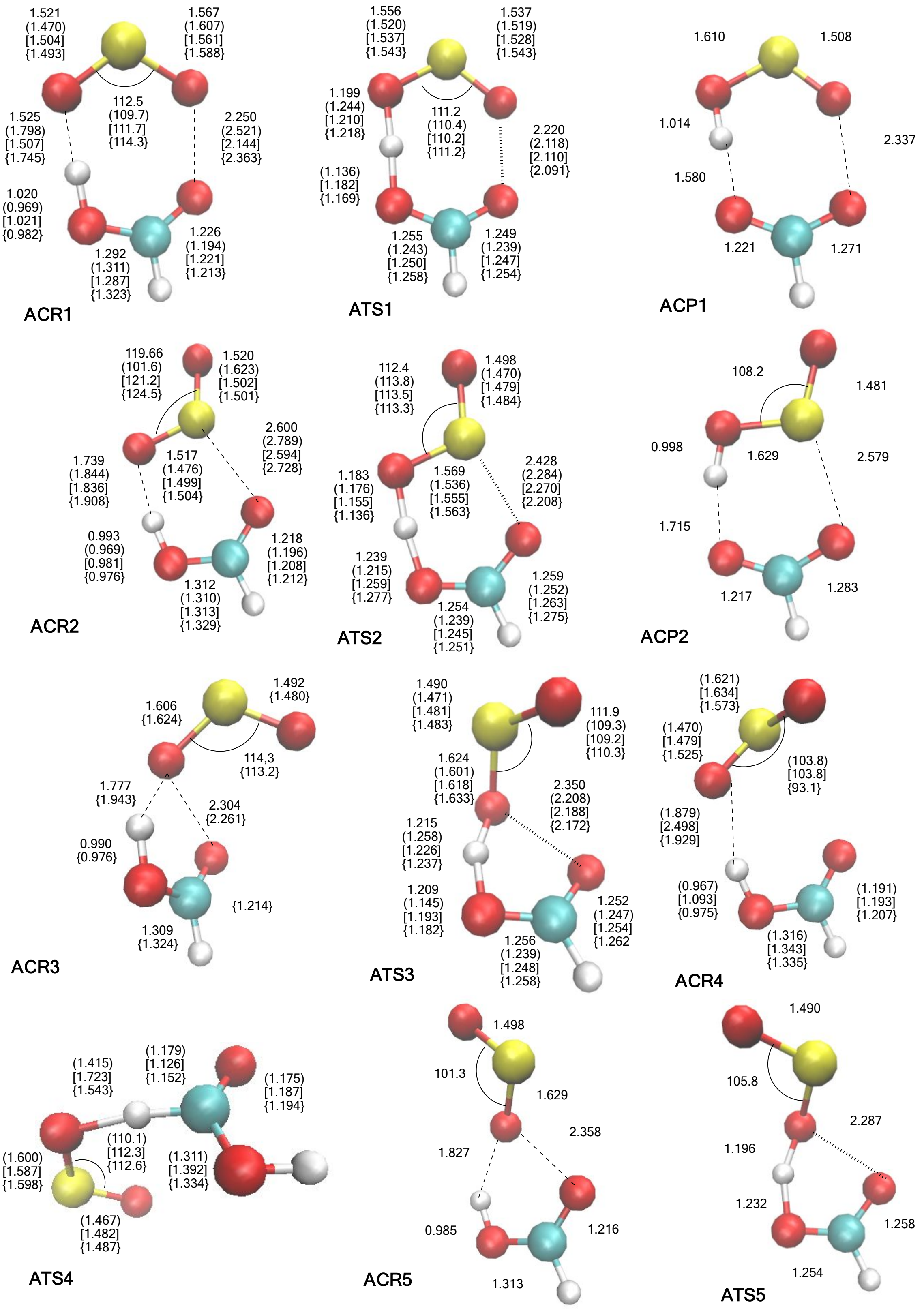
1.521 (1.470) [1.504] {1.493}
1.567 (1.607) [1.561] {1.588}
112.5 (109.7) [111.7] {114.3}
1.525 (1.798) [1.507] {1.745}
2.250 (2.521) [2.144] {2.363}
1.020 (0.969) [1.021] {0.982}
1.226 (1.194) [1.221] {1.213}
1.292 (1.311) [1.287] {1.323}
ACR1
1.556 (1.520) [1.537] {1.543}
1.537 (1.519) [1.528] {1.543}
1.199 (1.244) [1.210] {1.218}
111.2 (110.4) [110.2] {111.2}
2.220 (2.118) [2.110] {2.091}
(1.136) [1.182] {1.169}
1.255 (1.243) [1.250] {1.258}
1.249 (1.239) [1.247] {1.254}
ATS1
1.610
1.508
1.014
2.337
1.580
1.221
1.271
ACP1
119.66 (101.6) [121.2] {124.5}
1.520 (1.623) [1.502] {1.501}
2.600 (2.789) [2.594] {2.728}
1.739 (1.844) [1.836] {1.908}
1.517 (1.476) [1.499] {1.504}
0.993 (0.969) [0.981] {0.976}
1.218 (1.196) [1.208] {1.212}
1.312 (1.310) [1.313] {1.329}
ACR2
112.4 (113.8) [113.5] {113.3}
1.498 (1.470) [1.479] {1.484}
2.428 (2.284) [2.270] {2.208}
1.183 (1.176) [1.155] {1.136}
1.569 (1.536) [1.555] {1.563}
1.239 (1.215) [1.259] {1.277}
1.259 (1.252) [1.263] {1.275}
1.254 (1.239) [1.245] {1.251}
ATS2
108.2
1.481
0.998
1.629
2.579
1.715
1.217
1.283
ACP2
1.492 {1.480}
1.606 {1.624}
114,3 {113.2}
1.777 {1.943}
2.304 {2.261}
0.990 {0.976}
{1.214}
1.309 {1.324}
ACR3
1.490 (1.471) [1.481] {1.483}
111.9 (109.3) [109.2] {110.3}
1.624 (1.601) [1.618] {1.633}
2.350 (2.208) [2.188] {2.172}
1.215 (1.258) [1.226] {1.237}
1.209 (1.145) [1.193] {1.182}
1.252 (1.247) [1.254] {1.262}
1.256 (1.239) [1.248] {1.258}
ATS3
(1.621) [1.634] {1.573}
(1.470) [1.479] {1.525}
(103.8) [103.8] {93.1}
(1.879) [2.498] {1.929}
(0.967) [1.093] {0.975}
(1.191) [1.193] {1.207}
(1.316) [1.343] {1.335}
ACR4
(1.415) [1.723] {1.543}
(1.179) [1.126] {1.152}
(1.175) [1.187] {1.194}
(110.1) [112.3] {112.6}
(1.600) [1.587] {1.598}
(1.311) [1.392] {1.334}
(1.467) [1.482] {1.487}
ATS4
1.498
101.3
1.629
2.358
1.827
0.985
1.216
1.313
ACR5
1.490
105.8
2.287
1.196
1.232
1.258
1.254
ATS5

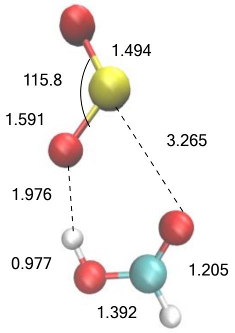

TS-ACR2ACR10

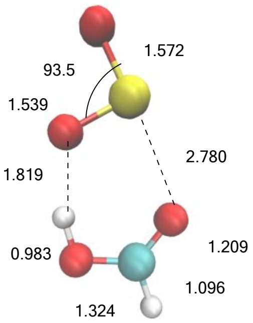

ACR10

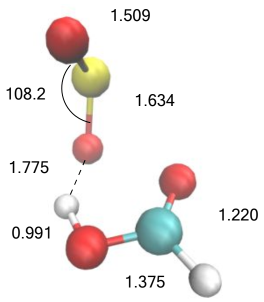

TS-ACR3ACR5

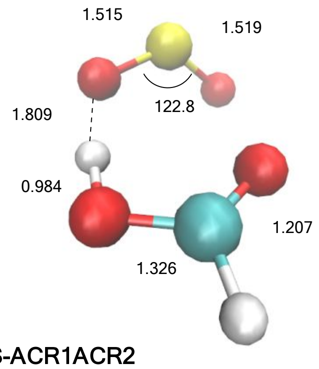

TS-ACR1ACR2

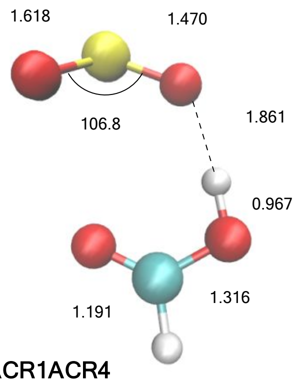

TS-ACR1ACR4

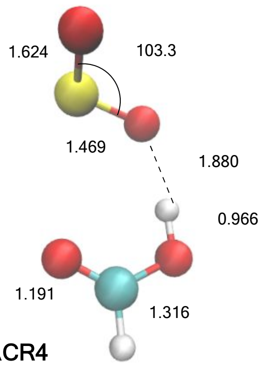

TS-ACR2ACR4

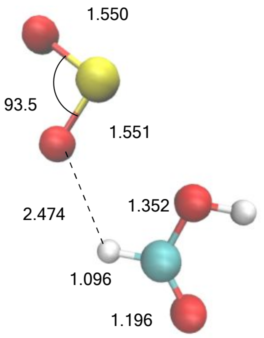

ACR6

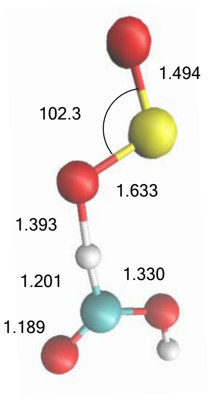

ATS6

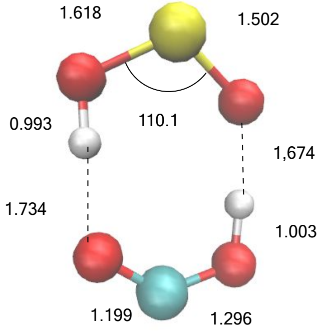

ACP3

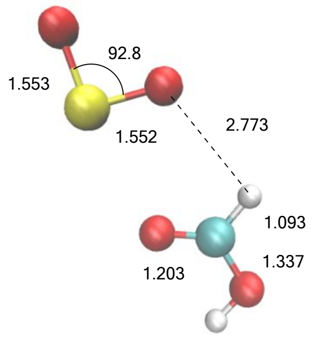

ACR7

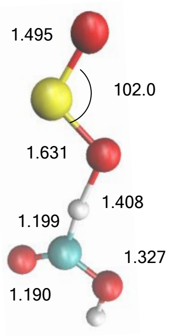

ATS7

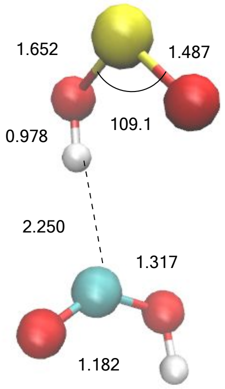

ACP4

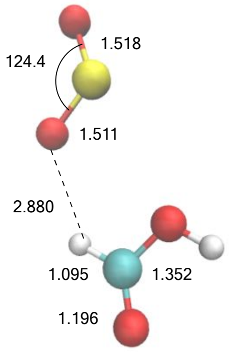

ACR8

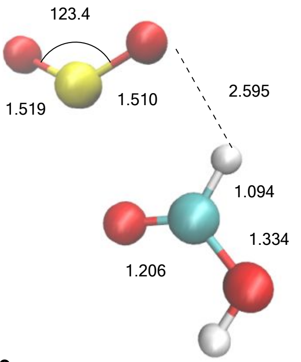

ACR9

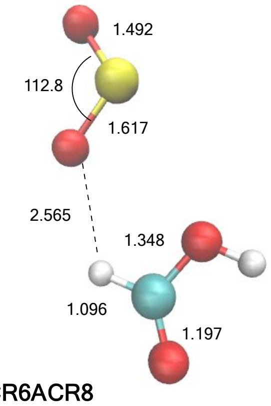

TA-SCR6ACR8

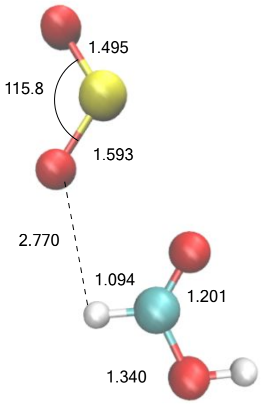

TS-ACR7ACR9

Figure S2. Main geometrical parameters of the stationary points for the reaction of $^3B_1$ and $^3A_2$ of SO_2 with HCOOH. Distances in Angstroms and angles in degrees. Values in plain text correspond to B3LYP optimized geometries. Values in parenthesis correspond to BH&HLYP optimized geometries. Values in square brackets correspond to M06-2X optimized geometries, and values in braces correspond to CCSD(T) optimized geometries.

In order to obtain accurate relative energies, we have carried out, for all optimized geometries, single point energy calculations at CCSD(T) method employing the aug-cc-pVTZ, aug-cc-pVQZ and complete CBS basis sets.[2–6] The results displayed in Table S1 show that the relative energies computed at CCSD(T)/CBS differ in less than 1.5 kcal·mol^{-1}. It has been pointed out previously, that quite large differences are found in the computed geometrical parameters of some pre-reactive complexes. Despite these differences, the relative energies computed at CCSD(T)/CBS over the pre-reactive complexes obtained with different theoretical approaches remain very small, indicating that the potential energy surface in these regions is very flat. Regarding the relative energies of the transition states, it is also worth mentioning that the entropic effect favors the acyclic (*hat*) structures with respect to the cyclic (*pcet*) transition states as expected. Thus, for instance, Table S1 shows that the energy gap between ATS1 and ATS4 is 4.96 kcal/mol and the Gibbs free energy gap is 3.51 kcal/mol, and a similar effect is shown in Table S3 for the reaction between triplet SO_2 and HNO_3 (see below).

Table S1. Relative energies, energies with zero-point corrections, enthalpies and free energies at 298 K and 1 atm for the reaction of $^3B_1$ and $^3A_2$ of SO_2 with HCOOH.

	**Method** [a]	**ΔE**	**Δ(E+ZPE)**	**ΔH**	**ΔG**
**SO_2 ($^3B_1$) + HCOOH**	CCSD(T)//B3LYP	0.00	0.00	0.00	0.00
	CCSD(T)//BH&HLYP	0.00	0.00	0.00	0.00
	CCSD(T)/M06-2X	0.00	0.00	0.00	0.00
	CCSD(T)// CCSD(T)	0.00	0.00	0.00	0.00
**SO_2 ($^3A_2$) + HCOOH**	CCSD(T)//B3LYP	1.29	1.01	1.05	0.89
**ACR1**	CCSD(T)//B3LYP	-3.01	-2.22	-2.43	7.75
	CCSD(T)//BH&HLYP	-3.29	-1.03	-1.15	8.19
	CCSD(T)/M06-2X	-3.25	-2.58	-2.77	7.25
	CCSD(T)// CCSD(T)	-3.93	-1.67	-1.79	7.55
**ACR2**	CCSD(T)//B3LYP	-6.13	-4.76	-4.79	5.02

	CCSD(T)//BH&HLYP	-5.04	-2.72	-2.90	7.06
	CCSD(T)/M06-2X	-6.68	-5.38	-5.30	4.16
	CCSD(T)// CCSD(T)	-6.77	-4.45	-4.63	5.33
**ACR3**	CCSD(T)//B3LYP	-0.29	0.68	0.78	9.98
	CCSD(T)// CCSD(T)	-0.32	1.80	1.80	10.88
**ACR4**	CCSD(T)//BH&HLYP	-2.75	-0.66	-0.65	8.18
	CCSD(T)/M06-2X	1.55	1.95	2.65	9.46
	CCSD(T)// CCSD(T)	-3.34	-1.25	-1.23	7.59
**ACR5**	CCSD(T)//B3LYP	-2.91	-1.94	-1.73	7.29
**ACR6**	CCSD(T)//B3LYP	-1.79	-1.63	-0.88	5.62
**ACR7**	CCSD(T)//B3LYP	-3.71	-3.32	-2.65	4.27
**ACR8**	CCSD(T)//B3LYP	-2.56	-2.12	-1.35	4.39
**ACR9**	CCSD(T)//B3LYP	-4.71	-3.70	-3.28	4.83
**ACR10**	CCSD(T)//B3LYP	-5.02	-4.38	-4.05	4.46
**TS-ACR1ACR2**	CCSD(T)//B3LYP	-3.78	-3.13	-3.29	6.27
**TS-ACR1ACR4**	CCSD(T)//BH&HLYP	-2.26	-0.25	-0.76	9.54
**TS-ACR2ACR4**	CCSD(T)//BH&HLYP	-2.52	-0.52	-1.02	9.26
**TS-ACR2ACR10**	CCSD(T)//B3LYP	-0.79	-0.81	-0.50	6.81
**TS-ACR3ACR5**	CCSD(T)//B3LYP	1.09	1.93	1.49	12.24
**TS-ACR5ACR10**	CCSD(T)//B3LYP	-3.67	-3.21	-3.30	6.33
**TS-ACR6ACR8**	CCSD(T)//B3LYP	2.08	1.61	2.31	7.76
**TS-ACR7ACR9**	CCSD(T)//B3LYP	0.68	0.41	0.96	7.60
**ATS1 (*pcet*)**	CCSD(T)//B3LYP	-0.09	-2.08	-2.58	8.24
	CCSD(T)//BH&HLYP	-0.86	-1.33	-2.31	9.48
	CCSD(T)/M06-2X	-0.69	-2.53	-3.09	7.90
	CCSD(T)// CCSD(T)	-0.79	-1.27	-2.25	9.54
**ATS2 (*pcet*)**	CCSD(T)//B3LYP	2.55	0.92	0.41	11.50
	CCSD(T)//BH&HLYP	1.20	1.05	0.00	12.29
	CCSD(T)/M06-2X	1.46	0.15	-0.50	10.82
	CCSD(T)// CCSD(T)	1.18	1.04	-0.02	12.28
**ATS3 (*pcet*)**	CCSD(T)//B3LYP	6.81	4.73	4.45	14.66
	CCSD(T)//BH&HLYP	6.01	5.50	4.68	16.02
	CCSD(T)/M06-2X	5.76	4.11	3.62	14.56

	CCSD(T)// CCSD(T)	5.71	5.21	4.38	15.73
**ATS4 (*hat*)**	CCSD(T)//BH&HLYP	4.10	3.06	2.47	12.99
	CCSD(T)/M06-2X	4.59	3.73	3.76	13.13
	CCSD(T)// CCSD(T)	4.62	3.57	2.99	13.51
**ATS5**	CCSD(T)//B3LYP	7.23	5.34	5.01	15.27
**ATS6**	CCSD(T)//B3LYP	7.42	5.27	5.27	14.58
**ATS7**	CCSD(T)//B3LYP	7.95	5.86	5.87	15.00
**ACP1**	CCSD(T)//B3LYP	-6.00	-6.39	-6.35	2.87
**ACP2**	CCSD(T)//B3LYP	-0.96	-1.09	-0.99	8.46
**ACP3**	CCSD(T)//B3LYP	-27.43	-26.75	-26.70	-17.37
**ACP4**	CCSD(T)//B3LYP	-15.93	-16.34	-15.33	-8.54
**HOSO + HCOO**	CCSD(T)//B3LYP	0.99	-0.76	-0.32	-1.76
**HOSO + HOCO**	CCSD(T)//B3LYP	-13.31	-14.49	-13.95	-15.85

a) CCSD(T)//B3LYP stands for CCSD(T)/CBS//B3LYP/aug-cc-pVTZ; CCSD(T)//BH&HYLYP stands for CCSD(T)/CBS//BH&HLYP/aug-cc-pVTZ; CCSD(T)//M06-2X stands for CCSD(T)/CBS//M06-2X/aug-cc-pVTZ; CCSD(T)//CCSD(T) stands for CCSD(T)/CBS//CCSD(T)/6-311+G(2df,2p) with zero point energies and thermodynamic corrections at BH&HLYP level.

Finally, for the ATS1, ATS2, and ATS3 electronic states we have calculated the topological properties of the bond critical points as displayed in Table S2

Table S2. Electron density ($\rho(r_b)$ in e·bohr^{-3}), Laplacian of the electron density ($\nabla^2\rho(r_b)$ in e·bohr^{-5}), the ellipticity (ε) and local energy density ($E(r_b)$ in hartree·bohr^{-3}) for all bond critical points (r_b) at ATS1, ATS2, and ATS3.[a]

ATS1

**bond**[a]	**$\rho(r_b)$**	**$\nabla^2\rho(r_b)$**	**ε**	**$E(r_b)$**
C1-O4	0.387301	-0.432125	0.067129	-0.651192
C1-H7	0.286947	-1.148121	0.021118	-0.314940
C1-O6	0.379732	-0.504339	0.066985	-0.629253
O6-H8	0.172979	-0.403865	0.006826	-0.187192
H8-O3	0.173519	-0.400551	0.012504	-0.188427
O3-S1	0.243464	0.262009	0.271431	-0.304334
S1-O2	0.253481	0.381588	0.260035	-0.319376

O2-O8	0.039943	0.161482	0.030705	0.004015
Ring	0.008118	0.035744	-1.387059	0.001600
		**ATS2**		
O2-S1	0.269587	0.631642	0.177743	-0.339805
S1-O3	0.240401	0.136726	0.319962	-0.303090
O3-H8	0.184844	-0.515085	0.006471	-0.217067
H8-O6	0.159899	-0.276160	0.002746	-0.153217
O6-C5	0.382064	-0.523224	0.073508	-0.635156
C5-H7	0.290419	-1.172902	0.023729	-0.320598
C5-O4	0.378305	-0.472461	0.056631	-0.628702
O4-S1	0.042546	0.105987	0.182670	-0.001743
Ring	0.014685	0.072804	-1.350355	0.002691
		**ATS3**		
C5-O6	0.385867	-0.446766	0.065751	-0.647867
C5-O4	0.382346	-0.548864	0.071710	-0.635337
C5-H7	0.289942	-1.176375	0.018360	-0.320914
O6-H8	0.176547	-0.406537	0.001717	-0.187569
H8-O3	0.169692	-0.333402	0.016590	-0.170541
O3-S1	0.215171	-0.035059	0.253335	-0.261405
S1-O2	0.273709	0.708168	0.167790	-0.343067
O4-O3	0.032215	0.124156	0.197728	0.003767
Ring	0.025957	0.139280	-1.604877	0.004960

a) See Figure 2 of the main text for atom numbering

The reaction of the $^3SO_2$ excited state with HNO_3.

The computed relative energies for the reaction between $^3B_1$ and $^3A_2$ electronic states of SO_2 with HNO_3 are collected in Table S3. Figure S3 shows how are connected the different stationary points (reactants, pre-reactive complexes, transition states, post-reactive complexes, and

product) .

Table S3. Relative energies, energies with zero-point corrections, enthalpies and free energies at 298 K and 1 atm for the reaction of $^3B_1$ and $^3A_2$ of SO_2 with HNO_3.[a]

	**ΔE**	**Δ (E+ZPE)**	**ΔH**	**ΔG**
**SO_2 ($^3B_1$) + HNO_3**	0.00	0.00	0.00	0.00
**SO_2 ($^3A_2$) + HNO_3**	1.29	1.01	1.05	0.89
**BCR1**	-2.04	-1.36	-1.18	8.19
**BCR2**	-5.01	-4.11	-3.78	5.02
**BCR3**	-4.25	-3.75	-3.23	4.72
**TS-BCR1BCR2**	5.05	5.40	5.48	14.39
**TS-BCR1BCR3**	-4.01	-3.69	-3.64	5.90
**BTS1**	5.23	3.44	3.09	14.07
**BTS2**	7.63	5.92	5.58	16.52
**BTS3**	11.49	9.56	9.36	19.74
**BTS4**	15.10	11.85	11.91	20.99
**BTS5**	9.97	8.06	7.88	18.09
**BTS6**	17.29	13.75	13.91	22.28
**BCP1**	-11.95	-13.59	-12.46	-6.48
**HOSO + NO_3**	-8.36	-11.19	-10.34	-11.95

a) Relative energies computed at CCSD(T)/CBS//B3LYP/aug-cc-pVTZ

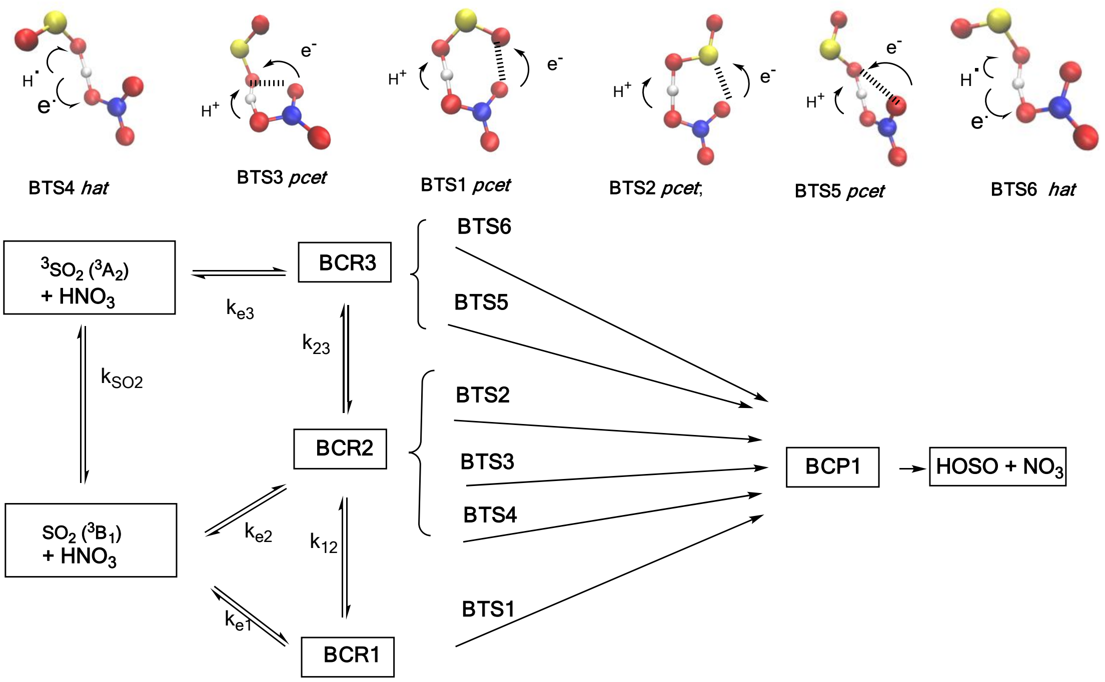

Figure S3: Scheme of the connection of the stationary points for the reaction between the electronic states $^3B_1$ and $^3A_2$ of SO_2 with HNO_3. All cartesian coordinates obtained with the different methods employed are reported below in Table S8.

In Figure S4 we have drawn electronic features of the most relevant natural orbitals of the transition states BTS1, BTS2, BTS3, and BTS4.

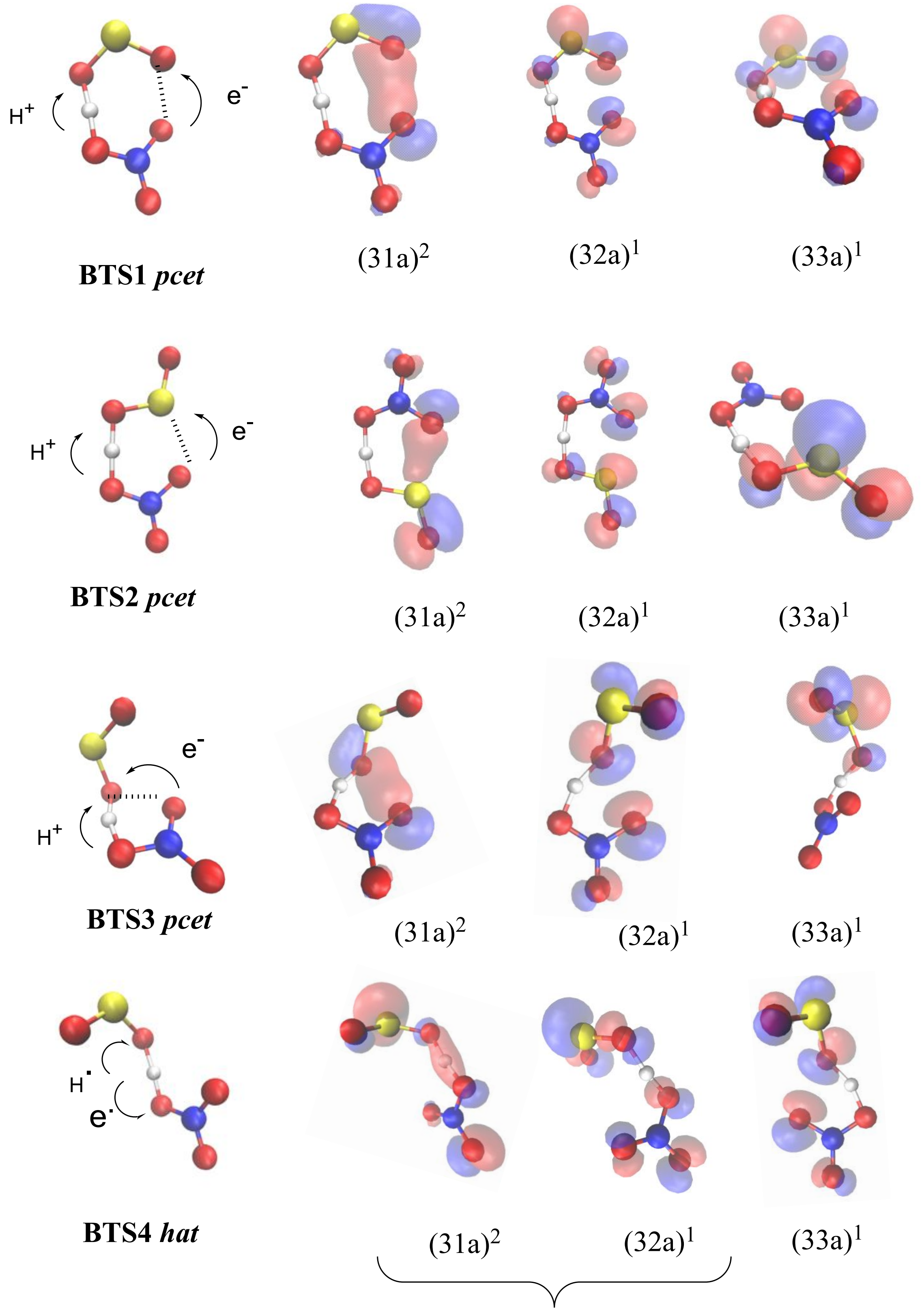

Figure S4. Picture of the natural orbitals involving the *pcet* and *hat* mechanisms for the SO_2 (a^3B_1) + HNO_3 reaction. The processes are described by the double occupied orbital

As pointed out in the main text, the processes take place by interaction of three electrons in two orbitals, (orbitals 31a and 32a with bonding and antibonding character, respectively) whereas orbital 33a (that corresponds to the $3b_1$ of $^3SO_2$) does not participate in the reaction and acts as spectator. Figure S4 shows that in BTS1 the electron is transferred from the oxygen atom of the acid to one oxygen atom of $^3SO_2$ while the acidic proton jumps to the other oxygen atom of sulfur dioxide in a seven-member ring structure. In BTS2 the electron transferred goes to the sulfur atom and the proton moves to one of the oxygen atoms of $^3SO_2$ in a six-member ring structure. BTS3 has a five-member ring structure where the electron is transferred to one of the oxygen atoms of $^3SO_2$ and the acidic proton jumps to the same oxygen atom. Finally, BTS4 has a *hat* mechanism and Figure S4 shows the features of the homolytic breaking and forming of the (O_2NO)-H-(OSO) bonds. In Figure S5 we have shown the most relevant geometrical parameters of the stationary points investigated.

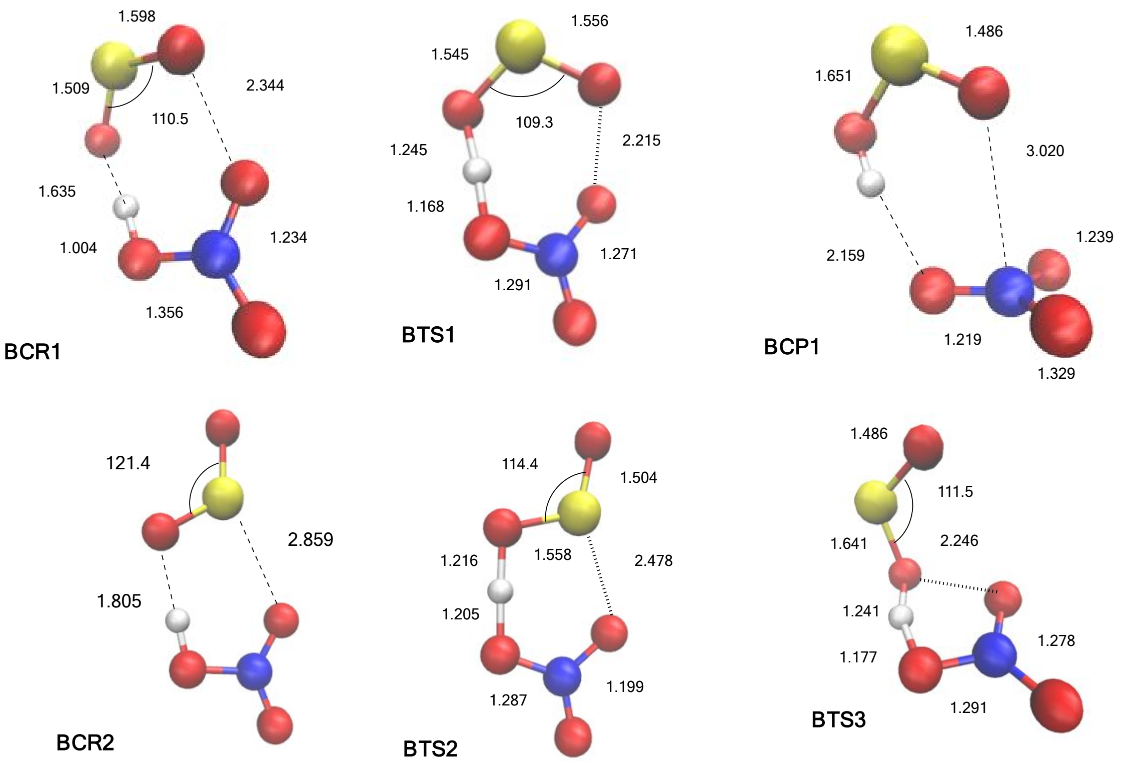

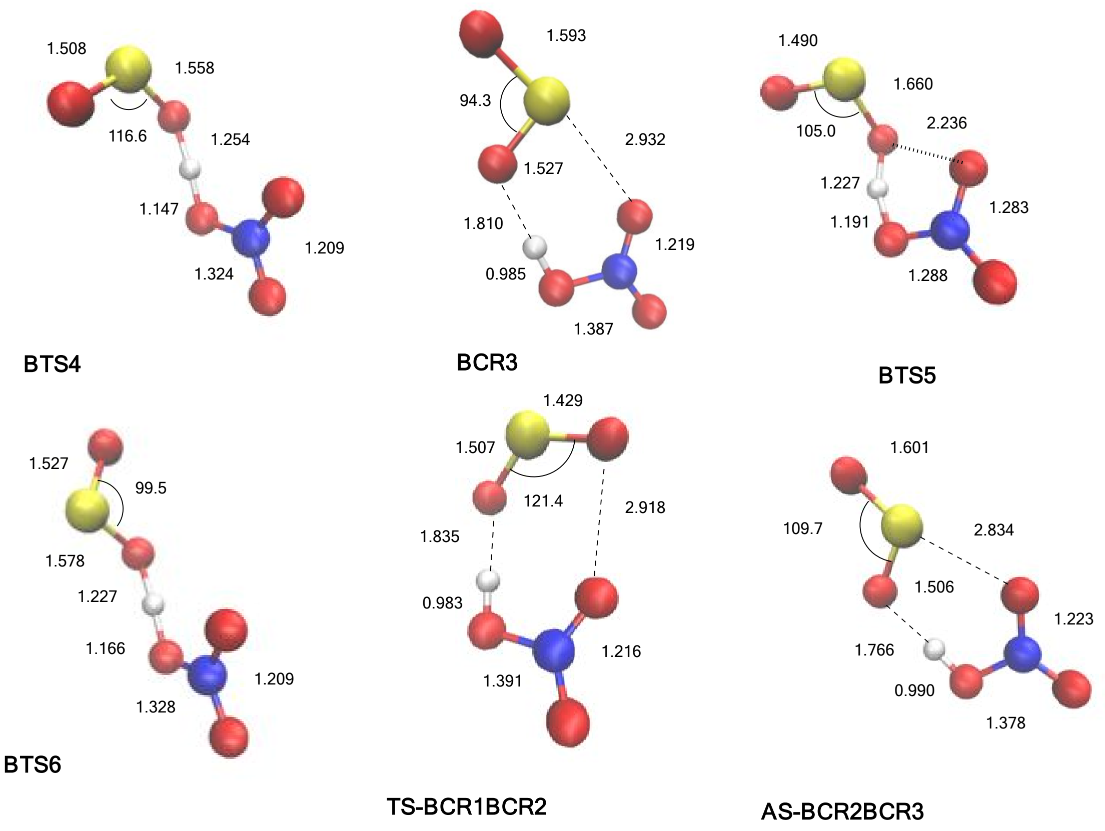

Figure S5. Main geometrical parameters of the stationary points for the reaction of $^3B_1$ and $^3A_2$ of SO_2 with HNO_3, optimized at B3LYP/aug-cc-pVTZ level of theory. Distances in Angstroms and angles in degrees.

The reaction of the $^3SO_2$ excited state with H_2O.

The reaction of SO_2 ($^3B_1$) with H_2O has been reported in a previous work,[1] and we address the reader to that article for a deep discussion. In this work, we have updated the previous work by re-optimizing the stationary points with the B3LYP/aug-cc-pVTZ level of theory, studying two additional reaction paths (CTS3 and CTS4) and performing a full kinetic study, with the aim of comparing the rate constants with those of the reactions of $^3SO_2$ with HCOOH and HNO_3. Table S4 contains the relative energies, whereas Figure S6 shows a scheme of the potential energy surface and Figure S7 the connection of the stationary points. Just mention that the lowest reaction path (via CTS1) has a *pcet* mechanism while the reaction paths via CTS2, CTS3, and CTS4 follow a *hat* mechanism, CTS2 lying lower than CTS3 and CTS4 because it is stabilized by a hydrogen bond. No stationary points were found for the reaction of $^3A_2$ of SO_2 with H_2O.

Table S4. Relative energies, energies with zero-point corrections, enthalpies at 298 K and free energies at 298 K for the reaction of $^3B_1$ and $^3A_2$ of SO_2 with H_2O.[a]

	ΔE	Δ(E+ZPE)	ΔH	ΔG
**SO_2 ($^3B_1$) + H_2O**	0.00	0.00	0.00	0.00
**SO_2 ($^3A_2$) + H_2O**	1.29	1.01	1.05	0.89
**CCR1**	-0.83	1.53	0.70	9.82
**CCR2**	-0.66	0.57	0.62	7.35
**CCR3**	-3.44	-2.57	-2.33	3.52
**CCR4**	-1.54	-0.71	-0.44	4.38
**CCR5**	-3.44	-2.15	-2.02	3.73
**TS-CCR2-CCR3**	-1.01	-0.20	-0.47	6.84
**TS-CCR3-CCR4**	-1.49	-0.74	-1.01	5.63
**TS-CCR1-CCR4**	0.65	1.37	1.17	7.87
**TS-CCR3-CCR5**	-0.06	0.65	0.42	7.63
**CTS1**	6.94	6.60	5.19	15.47
**CTS2**	9.64	8.08	6.94	16.52
**CTS3**	14.61	12.50	11.72	20.13
**CTS4**	14.78	12.83	12.05	20.29
**CCP1**	-1.90	-0.84	-1.39	6.75
**CCP2**	1.00	1.93	1.38	9.17
**HOSO + OH**	5.53	4.59	4.81	3.89

a) Relative energies computed at CCSD(T)/CBS//B3LYP/aug-cc-pVTZ

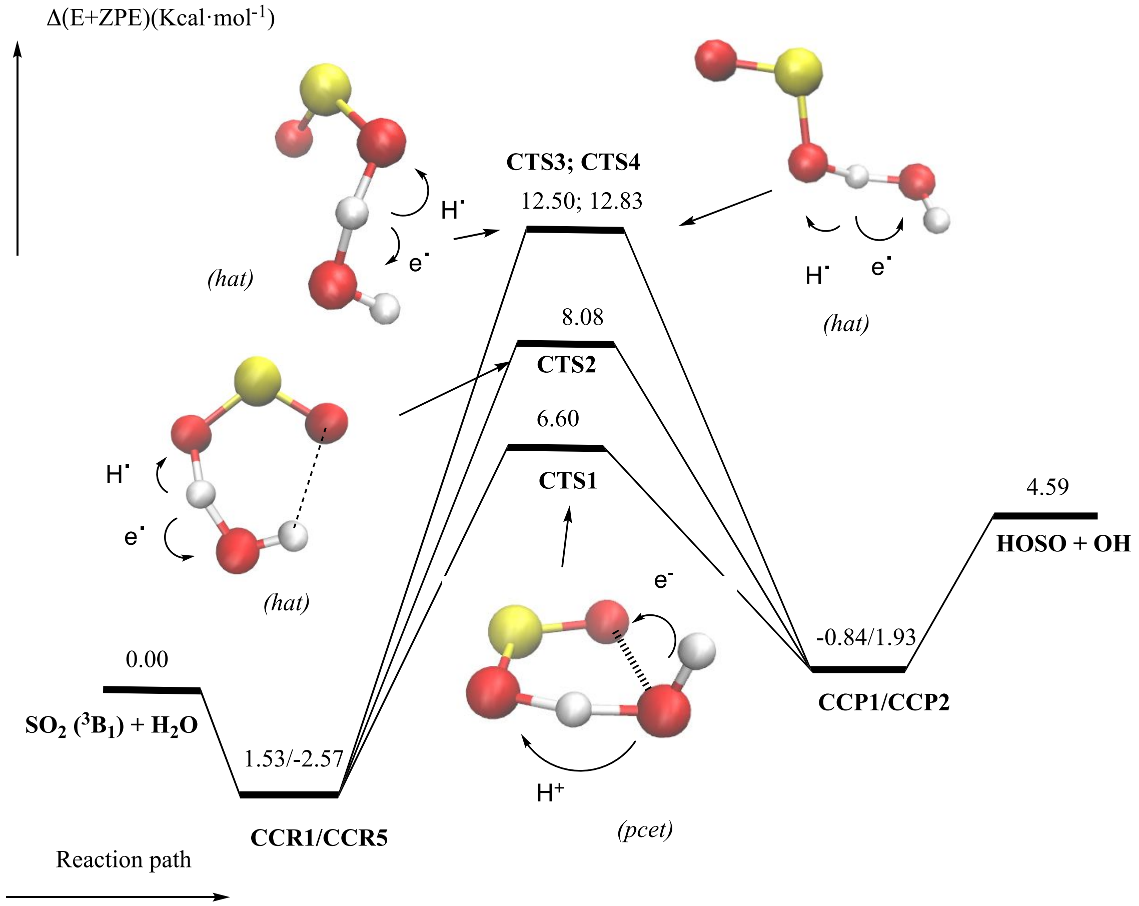

Figure S6. Schematic potential energy surface for the reaction of $^3SO_2$ with H_2O. Energies in Hartree including zero-point corrections.

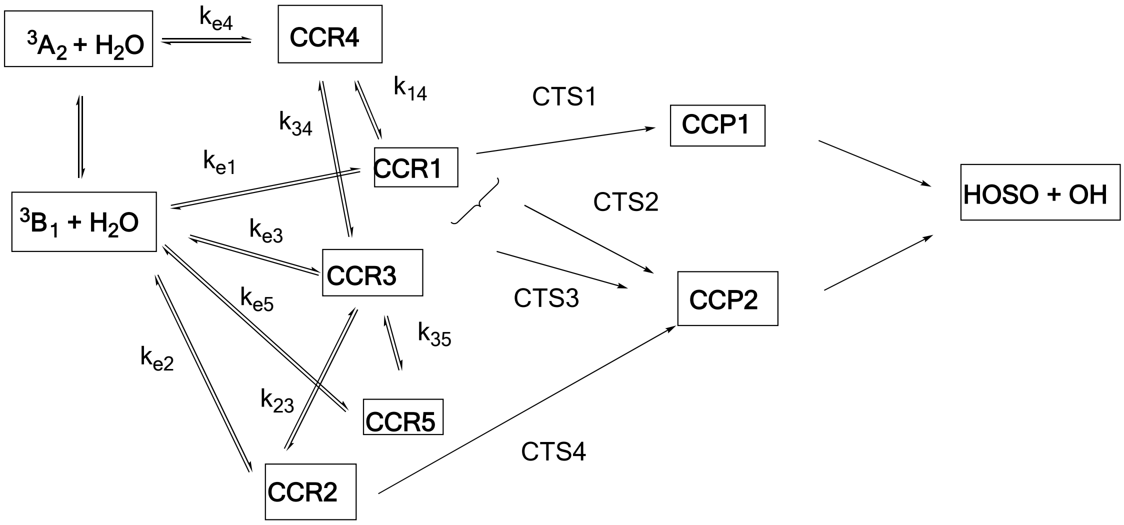

Figure S7: Scheme of the connection of the stationary points for the reaction between $^3SO_2$ with H_2O. All cartesian coordinates obtained with the different methods employed are reported below in Table S9.

Kinetic study.

Along this work we have shown that the reaction of $^3SO_2$ with HCOOH can lead to the formation of $HOSO^\bullet$ + $HCOO^\bullet$ radicals (reactions RS1) or $HOSO^\bullet$ + $HOCO^\bullet$ radicals (reaction RS2), whereas the reactions of $^3SO_2$ with HNO_3 produces $HOSO^\bullet$ + $NO_3^\bullet$ radicals (reaction RS3), and the reaction of $^3SO_2$ + H_2O forms $HOSO^\bullet$ + $OH^\bullet$ radicals (reaction RS4)

$$^3SO_2 + HCOOH \rightarrow HOSO^\bullet + HCOO^\bullet \quad (RS1)$$

$$^3SO_2 + HCOOH \rightarrow HOSO^\bullet + HOCO^\bullet \quad (RS2)$$

$$^3SO_2 + HNO_3 \rightarrow HOSO^\bullet + NO_3^\bullet \quad (RS3)$$

$$^3SO_2 + H_2O \rightarrow HOSO^\bullet + OH^\bullet \quad (RS4)$$

Figures S1, S3, and S7 show the complexity of these reactions, where the interaction between the reactants leads to the formation of several pre-reactive complexes before the transition states and the formation of the products. Moreover, several pre-reactive complexes are interconnected and therefore may have an impact in the rection kinetics. Therefore, as pointed out above, we have considered all steps shown in Figures S1, S3, and S7, and we have performed a numerical integration to calculate the corresponding rate constants. Regarding the unimolecular steps, we have employed conventional transition state theory (CTST) for the steps involving reorganization of the pre-reactive complexes, variational transition state theory (VTST) for the calculations at ground level, namely with pressure of 1 atm and different temperatures, and the RRKM approach for calculations at different temperatures and pressures. In all cases we have considered energies calculated at CCSD(T)/CBS level of theory and partition functions computed at B3LYP/aug-cc-pVTZ level of theory except for ATS4 where we have taken the partition functions at BH&HLYP/aug-cc-pVTZ level of theory. In the case of the VTST calculations we have calculated the hessian matrices and CCSD(T) energies of about 10 and 12 of each side of the potential energy surface and we have interpolated these values to the whole potential energy surface as described in Polyrate. In these calculations the tunneling parameter has been computed with the small curvature approach. In the case of the RRKM calculations we have considered that the reaction takes place in N_2 bath simulated with a Lennard-Jones potential with the following parameters σ = 4.07 Å and ε/κ = 248.6 for the reaction of $^3SO_2$ with HCOOH; σ = 4.05 Å and ε/κ = 252.0 for the

reaction of $^3SO_2$ with HNO3; and σ = 3.41 Å and ε/κ = 412.3 for the reaction of $^3SO_2$ with H_2O. These values have been deduced combining the rules for Lenard-Jones parameters . $\sigma_{AB} = \frac{1}{2}(\sigma_A + \sigma_B)$ and

$(\varepsilon/\kappa_B)_{AB} = [(\varepsilon/\kappa_B)_A \cdot (\varepsilon/\kappa_B)_B]^{1/2}$, where we have taken the σ and ε/κ values of 4.11 and 336 for SO_2,[7] 4.04 and 183.9 for HCOOH,[8] 3.98 and 189 for HNO_3,[9] and 2.71 and 506 for H_2O.[10] The numerical integration has been performed by an ad-hoc made program written in python.[11]

Table 3 of the main text contains the rate constants calculated at different heights in the Earth's atmosphere, and in Table S5 we have collected the rate constants at different temperatures at ground level.

Table S5. Rate constants, in $cm^3 \cdot molecule^{-1} \cdot s^{-1}$, for the reactions of $^3SO_2$ with HCOOH (k_{RS1}, k_{RS1}, and k_{RS1}+ k_{RS2}), with HNO_3 (k_{RS3}), and with H_2O (k_{RS4}), at different temperatures T (in K) at ground level in the Earth's atmosphere. The branching ratios for RS1 ($\%_{RS1}$) and RS2 ($\%_{RS2}$) for the $^3SO_2$ with HCOOH reaction are also given.

**T**	k_{RS1}	k_{RS2}	$k_{RS1}+k_{RS2}$	$\%_{RS1}$	$\%_{RS2}$	k_{RS3}	k_{RS4}
**200.0**	$1.87\cdot10^{-12}$	$4.64\cdot10^{-17}$	$1.87\cdot10^{-12}$	100.00	0.00	$4.31\cdot10^{-18}$	$3.36\cdot10^{-18}$
**220.0**	$1.15\cdot10^{-12}$	$7.79\cdot10^{-17}$	$1.15\cdot10^{-12}$	99.99	0.01	$6.79\cdot10^{-18}$	$6.80\cdot10^{-18}$
**240.0**	$7.74\cdot10^{-13}$	$1.25\cdot10^{-16}$	$7.74\cdot10^{-13}$	99.98	0.02	$1.09\cdot10^{-17}$	$1.30\cdot10^{-17}$
**260.0**	$5.61\cdot10^{-13}$	$1.95\cdot10^{-16}$	$5.62\cdot10^{-13}$	99.97	0.03	$1.74\cdot10^{-17}$	$2.33\cdot10^{-17}$
**280.0**	$4.32\cdot10^{-13}$	$2.95\cdot10^{-16}$	$4.32\cdot10^{-13}$	99.93	0.07	$2.73\cdot10^{-17}$	$4.03\cdot10^{-17}$
**288.8**	$3.90\cdot10^{-13}$	$3.52\cdot10^{-16}$	$3.90\cdot10^{-13}$	99.91	0.09	$3.28\cdot10^{-17}$	$5.05\cdot10^{-17}$
**298.1**	$3.54\cdot10^{-13}$	$4.21\cdot10^{-16}$	$3.54\cdot10^{-13}$	99.88	0.12	$3.97\cdot10^{-17}$	$6.35\cdot10^{-17}$
**300.0**	$3.44\cdot10^{-13}$	$4.38\cdot10^{-16}$	$3.44\cdot10^{-13}$	99.87	0.13	$4.15\cdot10^{-17}$	$6.65\cdot10^{-17}$
**320.0**	$2.86\cdot10^{-13}$	$6.39\cdot10^{-16}$	$2.87\cdot10^{-13}$	99.78	0.22	$6.16\cdot10^{-17}$	$1.06\cdot10^{-16}$
**340.0**	$2.46\cdot10^{-13}$	$9.20\cdot10^{-16}$	$2.476\cdot10^{-13}$	99.63	0.37	$8.90\cdot10^{-17}$	$1.62\cdot10^{-16}$
**350.0**	$2.32\cdot10^{-13}$	$1.10\cdot10^{-15}$	$2.33\cdot10^{-13}$	99.53	0.47	$1.06\cdot10^{-16}$	$1.51\cdot10^{-17}$

Our calculations predict that the rate constants for reaction RS1 + RS2 ($^3SO_2$ + HCOOH) are between four and five orders of magnitude greater than RS3 ($^3SO_2$ + HNO_3) and RS4 ($^3SO_2$ + H_2O), in line with the values discussed in the main text. Moreover, the reaction of $^3SO_2$ with HCOOH produces almost exclusively HOSO$^\bullet$ + HCOO$^\bullet$ radicals (reaction

RS1). Indeed, $^3SO_2$ abstracts the acidic hydrogen instead of the formyl hydrogen, which is the opposite to what would be expected, since the bond dissociation energy (BDE) of the C-H bond (96.2 ± 07 kcal·mol^{-1}) is smaller than the BDE of the O-H bond (112.2 ± 3.1 kcal·mol^{-1}).[12] This behavior is consistent with previous findings for the reaction of formic acid with hydroxyl radical.[13]

Cartesian coordinates

Table S6. Cartesian coordinates (in Angstroms) of the stationary points for the reaction of $^3B_1$ and $^3A_2$ of SO_2 with HCOOH, computed at B3LYP/aug-cc-pVTZ level of theory.

ACR1

C	2.078349	-0.119530	0.362006
O	1.472614	-1.128780	0.020283
H	3.158279	-0.200646	0.542499
O	1.624079	1.074671	0.552456
H	0.616706	1.142641	0.387961
O	-0.885057	1.258515	0.151408
S	-1.648867	0.026103	-0.306382
O	-0.726047	-1.232517	-0.445498

ATS1

C	-0.291401	-0.179085	-0.269553
O	-0.815885	-1.257135	-0.619101
H	0.794142	-0.269612	-0.088835
O	-0.791011	0.958667	-0.096389
H	-1.976035	1.059914	-0.288092
O	-3.151611	1.213862	-0.464243
S	-3.930214	-0.052917	-0.924301
O	-2.994437	-1.266133	-1.044515

ACP1

C	2.088721	-0.116928	0.364486
O	1.624769	-1.246118	0.010856
H	3.183491	-0.259315	0.536753
O	1.583218	0.982255	0.528517
H	0.037856	1.227837	0.303411
O	-0.955147	1.372161	0.159127
S	-1.675104	0.012786	-0.314624
O	-0.672802	-1.110627	-0.396136

ACR2

O	-2.522112	0.675053	-0.270844
S	-1.058091	0.301797	-0.102804
O	-0.095226	1.300782	0.511476
H	1.468912	0.543525	0.533109
O	2.255260	-0.058289	0.454614

C	1.895051	-1.200121	-0.080414
H	2.738720	-1.891382	-0.181883
O	0.773917	-1.512879	-0.441230

ATS2

O	-0.726923	-0.113812	0.852556
S	0.670949	-0.646019	0.933258
O	1.667789	0.388600	1.563369
H	2.727581	-0.136882	1.578736
O	3.758725	-0.821333	1.523063
C	3.486376	-1.934938	1.014136
H	4.308614	-2.658240	0.897191
O	2.369534	-2.350262	0.607301

ACP2

O	-2.410298	0.724913	-0.220868
S	-1.019494	0.223265	-0.127455
O	-0.101930	1.392050	0.540316
H	0.818750	1.008975	0.581681
O	2.157015	-0.053707	0.438369
C	1.876067	-1.130159	-0.056126
H	2.652262	-1.917341	-0.207025
O	0.764046	-1.605097	-0.485410

ACR3

C	-2.125749	0.321775	-0.127131
O	-1.302267	0.837235	0.607289
O	-1.994094	-0.831318	-0.731759
H	-1.107740	-1.179773	-0.459726
O	0.287420	-0.830450	0.583418
S	1.697847	-0.233677	0.098263
H	-3.080568	0.813155	-0.340794
O	1.607663	1.115956	-0.531262

ATS3

C	-2.037755	0.285264	-0.135622
O	-1.383686	0.865625	0.760461
O	-1.701528	-0.769119	-0.729735
H	-0.648432	-0.983308	-0.176515
O	0.246004	-0.822812	0.629642
S	1.657734	-0.266508	0.050064
H	-2.991156	0.763428	-0.404873
O	1.507006	1.072859	-0.586106

ACR5

C	2.341960	-0.083143	-0.210109
O	1.506505	-0.961463	-0.116643
O	2.135782	1.197402	-0.005410
H	1.187203	1.298914	0.241053
O	-0.306364	0.333980	0.656686

S	-1.420079	-0.462287	-0.225668
H	3.379015	-0.298771	-0.487087
O	-2.642063	0.367178	0.024735

ATS5

C	2.183102	0.017192	-0.253925
O	1.609214	-1.069002	0.016544
O	1.689101	1.157948	-0.091423
H	0.601576	0.828330	0.383267
O	-0.199701	0.065085	0.836451
S	-1.400762	-0.452653	-0.169353
H	3.197012	-0.073792	-0.667901
O	-2.409241	0.644064	-0.19684

TS-ACR2BCR10

C	-0.120479	-0.038407	-0.057556
O	-0.156278	0.392930	1.067381
O	0.986904	-0.343794	-0.726381
H	1.746243	-0.153309	-0.142292
O	2.727171	0.450943	1.463066
S	2.451634	0.886061	2.968890
H	-1.011216	-0.231246	-0.666465
O	3.663339	1.076509	3.822195

ACR10

C	-1.768807	-0.473308	-1.600410
O	-1.830267	-0.067789	-0.463160
O	-0.659507	-0.760875	-2.263085
H	0.106476	-0.587062	-1.671459
O	1.107978	-0.116232	-0.228337
S	0.444699	0.349832	1.080131
H	-2.653541	-0.651334	-2.222277
O	1.818025	0.530500	1.824257

TS-ACR3ACR5

C	-0.000470	-0.000576	-0.000275
O	-0.001954	-0.003641	1.219738
O	1.059444	0.002596	-0.762240
H	1.837963	0.016099	-0.148214
O	2.281403	-0.107540	1.565830
S	2.460594	1.223631	2.496605
H	-0.938215	-0.000711	-0.565375
O	2.277208	2.439147	1.622224

TS-ACR1ACR2

C	0.038745	-0.040625	-0.033239
O	0.036539	-0.523596	1.072489
H	0.956072	0.147077	-0.604707
O	-1.030435	0.331614	-0.723321
H	-1.832096	0.138098	-0.185739

O	-3.131223	-0.287823	0.998941
S	-2.836680	-0.992883	2.307085
O	-2.027049	-0.354717	3.422885

ACR6

C	0.854298	-0.671049	2.056166
O	1.373766	-0.989428	3.085171
O	1.469623	0.016382	1.068345
H	2.381079	0.192387	1.351570
H	-0.180217	-0.885397	1.765363
O	-1.183835	-0.205843	-0.391747
S	-0.317063	0.564402	-1.422350
O	-1.457425	0.637845	-2.470366

ATS6

C	0.435718	-0.114550	0.071913
O	0.308344	-0.659138	1.121217
O	1.513242	0.497467	-0.412429
H	2.233688	0.443963	0.238385
H	-0.449554	-0.041445	-0.737837
O	-1.404537	0.108042	-1.740681
S	-0.872882	0.891954	-3.070700
O	-2.088550	0.905069	-3.939131

ACP3

C	1.574499	-0.754533	1.848817
O	0.617459	-1.414051	1.554060
O	1.983962	0.403639	1.435902
H	1.352113	0.784944	0.756287
H	-0.538050	-1.100176	0.299981
O	-1.149187	-0.875053	-0.450095
S	-1.088125	0.694857	-0.836980
O	0.190832	1.300587	-0.334323

ACR7

C	2.075748	0.394927	0.709313
O	1.143775	-0.205329	1.177474
O	3.270996	0.520916	1.296093
H	3.245061	0.050642	2.144836
H	2.064115	0.913051	-0.253755
O	-0.493681	0.409038	-1.200021
S	-1.365444	-0.404305	-0.206103
O	-2.602447	-0.370343	-1.144097

ATS7

C	0.428076	-0.116486	0.077074
O	0.308484	-0.662344	1.128542
O	1.498225	0.492808	-0.418176
H	2.224308	0.436308	0.226152
H	-0.457892	-0.038748	-0.726507

O	-1.396958	0.132418	-1.760881
S	-2.837794	-0.533827	-1.384467
O	-3.642886	-0.245409	-2.611201

ACP4

C	1.814377	-0.053926	0.937804
O	1.744721	-0.804577	1.848281
O	2.408166	1.114329	0.802440
H	2.836710	1.374721	1.641018
H	0.451509	-0.621219	-0.760075
O	-0.198772	-0.776899	-1.474209
S	-1.619647	-0.028100	-1.089112
O	-1.362157	0.993074	-0.038323

ACR8

C	0.022028	-0.002670	2.463620
O	0.203878	-0.005006	3.645948
O	1.006940	-0.009801	1.537415
H	1.855381	-0.016935	2.008517
H	-0.952966	0.005415	1.965735
O	-1.409368	0.011181	-0.877454
S	-0.020692	0.002029	-1.473778
O	0.272251	0.002648	-2.963322

ACR9

C	1.449142	0.000697	-1.368408
O	1.575298	0.002041	-0.168775
O	2.463982	0.001215	-2.233687
H	3.296598	0.002906	-1.734290
H	0.485592	-0.001241	-1.886573
O	-1.666546	-0.002281	-0.436900
S	-0.973786	-0.000469	0.905364
O	-1.717622	-0.000543	2.230124

TS-ACR6ACR8

C	-0.022740	0.001478	0.004262
O	-0.001781	-0.002466	1.201053
O	1.070705	-0.004766	-0.784262
H	1.852068	-0.012931	-0.208678
H	-0.925012	0.010622	-0.617110
O	-1.049940	0.011319	-3.179464
S	0.151613	0.005253	-4.261512
O	-0.337899	0.008694	-5.670729

TS-ACR7ACR9

C	0.047106	-0.000457	0.009459
O	0.089432	0.000222	1.209998
O	1.119361	-0.000098	-0.794108
H	1.914815	0.000794	-0.238067
H	-0.873499	-0.001460	-0.581773

O	-2.778658	-0.003247	1.429163
S	-2.628942	-0.001611	3.014794
O	-3.908743	-0.002611	3.788450

Table S7. Cartesian coordinates (in Angstroms) of the stationary points for the reaction of $^3B_1$ of SO_2 with HCOOH, computed at BH&HLYP/aug-cc-pVTZ level of theory.

ACR1

C	2.218817	-0.101971	0.413284
O	1.524777	-1.004323	0.054737
H	3.285382	-0.211169	0.598248
O	1.824768	1.128081	0.635934
H	0.873523	1.198269	0.463863
O	-0.885486	1.129733	0.098356
S	-1.765586	0.038272	-0.344908
O	-0.915036	-1.316429	-0.496140

ATS1

C	-0.322697	-0.178240	-0.261228
O	-0.863515	-1.235992	-0.611326
H	0.747881	-0.281013	-0.075093
O	-0.812285	0.951873	-0.096063
H	-1.928394	1.054790	-0.282822
O	-3.149912	1.186869	-0.479580
S	-3.891251	-0.057033	-0.942955
O	-2.936279	-1.233694	-1.045961

ACR2

O	-2.584554	1.075752	-0.116933
S	-1.227992	0.187062	-0.187658
O	-0.237849	1.110658	0.399115
H	1.499873	0.505263	0.523570
O	2.309428	-0.025490	0.480458
C	2.029676	-1.191099	-0.048605
H	2.915673	-1.817665	-0.113635
O	0.948978	-1.542292	-0.419045

ATS2.

O	-0.653249	-0.201972	0.829067
S	0.738024	-0.661918	0.942344
O	1.685241	0.374380	1.565036
H	2.750879	-0.122872	1.589239
O	3.745007	-0.818264	1.518284
C	3.445854	-1.909980	1.015246
H	4.235223	-2.649991	0.884114
O	2.315666	-2.282269	0.626281

ATS3

C	-1.957225	0.283894	-0.129423

O	-1.314565	0.740764	0.837206
O	-1.634629	-0.719748	-0.782029
H	-0.653633	-0.984289	-0.252828
O	0.215018	-0.840373	0.646024
S	1.567771	-0.072390	0.267606
H	-2.862819	0.828551	-0.386696
O	1.288269	0.909020	-0.792542

ACR4

O	-0.894965	-0.559369	-0.963267
S	-1.366460	0.763051	-0.529035
O	-1.697110	0.558707	1.045040
H	3.304520	-0.556613	0.501972
C	2.270552	-0.363781	0.224486
O	1.745066	-1.412693	-0.372103
H	0.827956	-1.221335	-0.610028
O	1.707565	0.663492	0.439509

ATS4

O	0.607401	-0.270198	0.030686
S	0.079074	-0.018976	1.376356
O	1.264733	-0.065104	2.450372
H	2.482320	-0.284051	1.762795
C	3.409634	-0.603540	1.107934
O	3.377534	-1.901826	0.929534
H	4.118386	-2.168408	0.381350
O	4.202885	0.173336	0.725041

TS-ACR1ACR4

C	-0.008716	-0.001797	0.013095
O	-0.014231	-0.042726	1.203532
H	0.898865	0.018136	-0.586469
O	-1.076856	0.025185	-0.754342
H	-1.867393	0.001768	-0.198380
O	-3.215094	-0.093217	1.081187
S	-3.156914	-0.125769	2.549805
O	-2.031849	0.947369	2.997481

TS-ACR2ACR4

C	-0.006399	0.007279	0.006649
O	-0.015388	0.034580	1.197230
H	0.902023	-0.001541	-0.591760
O	-1.072187	-0.016468	-0.765328
H	-1.867712	-0.007517	-0.216789
O	-3.239734	0.008300	1.068630
S	-3.021870	0.125764	2.516743
O	-3.315339	1.695334	2.814371

Table S8. Cartesian coordinates (in Angstroms) of the stationary points for the reaction of $^3B_1$ of SO_2 with HCOOH, computed at M06-2X/aug-cc-pVTZ level of theory.

ACR1

C	1.854856	-.049781	-.886172
O	1.743600	-.043292	.330151
H	2.862955	-.022062	-1.317116
O	.921895	-.089698	-1.772061
H	.002160	-.199930	-1.340893
O	-1.309745	-.429589	-.635257
S	-1.445851	.092668	.768897
O	-.056353	.424374	1.397173

ACR2

O	-2.558212	.646669	-.282286
S	-1.102609	.315933	-.111363
O	-.145611	1.306732	.479111
H	1.523974	.545844	.552457
O	2.285735	-.066751	.469576
C	1.890161	-1.194290	-.077848
H	2.718265	-1.901274	-.188293
O	.764263	-1.456683	-.428364

ACR4

O	-1.037963	1.106531	-.085412
S	-1.848795	-.122518	.052130
O	-1.373813	-.744002	1.486836
H	1.391326	.955630	-.648718
C	1.969320	.066001	-.384638
O	1.159085	-1.002065	-.300607
H	1.702360	-1.763577	-.051400
O	3.146285	.029800	-.192345

ATS1

C	.002015	.000631	.000192
O	.003842	.018184	1.246734
H	1.015069	.005235	-.432390
O	-.954815	-.025747	-.804015
H	-2.019943	-.182342	-.314983
O	-3.100021	-.386166	.191648
S	-3.218994	.160504	1.623732
O	-1.831144	.441355	2.198456

ATS2

O	-.656605	-.205929	.809924
S	.742208	-.668369	.940142
O	1.686760	.387247	1.581349
H	2.734035	-.100065	1.598822
O	3.765025	-.818911	1.518367
C	3.449855	-1.913348	1.015275
H	4.235663	-2.668629	.873971
O	2.305703	-2.284882	.631762

ATS3

C	-1.919310	.297784	-.134944
O	-1.272944	.737412	.846105
O	-1.630296	-.730232	-.781526
H	-.635684	-1.023695	-.191753
O	.206245	-.866807	.685708
S	1.541740	-.107928	.177054
H	-2.804091	.885091	-.410751
O	1.162527	.953805	-.782576

ATS4

O	.840302	-.212238	.007271
S	.118314	-.232281	1.301638
O	1.104642	-.186325	2.543523
H	2.685371	-.109010	1.860592
C	3.426882	-.523758	1.121577
O	3.250059	-1.835922	1.012117
H	3.876857	-2.175054	.355807
O	4.239540	.135820	.561544

Table S9. Cartesian coordinates (in Angstroms) of the stationary points for the reaction of $^3B_1$ of SO_2 with HCOOH, computed at CCSD(T)/6-311+G(2df,2p) level of theory.

ACR1

C	2.178607	-0.112226	0.400306
O	1.477675	-1.027047	0.019898
H	3.250026	-0.233684	0.589835
O	1.791959	1.129870	0.640403
H	0.829492	1.197679	0.456951
O	-0.879696	1.208635	0.103611
S	-1.670057	0.014505	-0.321841
O	-0.816848	-1.317269	-0.465787

ATS1

C	1.932169	0.482349	0.000000
O	1.698114	-0.750128	0.000000
H	3.009179	0.707756	0.000000

O	1.154124	1.470470	0.000000
H	0.011991	1.220944	0.000000
O	-1.189141	1.019639	0.000000
S	-1.558284	-0.478640	0.000000
O	-0.301584	-1.359693	0.000000

ACR2

O	-0.001373	-0.002069	0.006641
S	-0.004760	0.000455	1.507791
O	1.233013	0.002662	2.362529
H	0.707127	-0.000390	4.196810
O	0.239204	-0.001527	5.053166
C	-1.068800	-0.000125	4.818269
H	-1.632114	-0.000824	5.755747
O	-1.593089	0.001819	3.725506

ATS2

C	-0.004897	0.010137	0.000010
H	0.008457	0.079664	1.094942
O	1.083638	0.039825	-0.615060
O	-1.175391	-0.093403	-0.494558
S	-1.170027	-0.245620	-2.697054
H	0.805356	-0.041085	-1.859751
O	-1.923896	-0.404232	-3.965954
O	0.373726	-0.119019	-2.908007

ACR3

C	-2.015991	0.348908	-0.116285
O	-1.278876	0.696864	0.784019
O	-1.888712	-0.757375	-0.833345
H	-1.098328	-1.215982	-0.490424
O	0.331555	-0.889945	0.784320
S	1.599949	-0.149437	0.090327
H	-2.886351	0.933413	-0.425823
O	1.219266	1.046456	-0.694490

ATS3

C	-1.924281	0.286887	-0.150108
O	-1.273066	0.750318	0.826172
O	-1.628569	-0.757721	-0.786454
H	-0.649535	-1.026807	-0.181808
O	0.190915	-0.849183	0.708207
S	1.546557	-0.146848	0.129179
H	-2.813966	0.859456	-0.433205
O	1.200133	1.029326	-0.704665

ACR4

O	-0.942636	-0.454688	-1.116486
S	-1.324531	0.858430	-0.443586
O	-1.384111	0.207729	0.987771

H	3.222743	-0.557236	0.607582
C	2.223742	-0.326811	0.224096
O	1.618175	-1.441649	-0.191912
H	0.732858	-1.197527	-0.519025
O	1.750886	0.783212	0.188134

ATS4

O	0.765245	-0.256859	0.010965
S	0.125293	-0.085992	1.341857
O	1.184789	-0.095700	2.538293
H	2.537475	-0.243509	1.810340
C	3.386362	-0.574520	1.107334
O	3.325287	-1.899044	0.962711
H	4.038974	-2.163490	0.362972
O	4.178534	0.180344	0.629598

Table S10. Cartesian coordinates (in Angstroms) of the stationary points for the reaction of $^3B_1$ and $^3A_2$ of SO_2 with HNO_3, computed at B3LYP/aug-cc-pVTZ level of theory.

BCR1

O	0.509064	-0.381856	-0.009433
S	0.310063	0.301006	1.421006
O	1.236757	-0.319616	2.437033
H	2.480533	-1.248515	1.923685
O	3.289627	-1.788123	1.674497
N	2.965097	-2.496452	0.564720
O	3.788151	-3.269164	0.171577
O	1.859173	-2.296696	0.056212

BCR2

O	-0.089695	-0.007487	-3.226610
S	0.004934	-0.003234	-1.705533
O	1.344248	0.004296	-0.998936
H	1.101553	0.006900	0.790793
O	0.951231	0.007997	1.767344
N	-0.418622	0.001860	1.934946
O	-0.788156	0.002282	3.069452
O	-1.101452	-0.003005	0.921538

BCR3

N	-1.551467	-0.889151	-0.984017
O	-1.473052	-0.292101	0.075596
O	-0.372009	-1.047236	-1.696270
H	0.315670	-0.612460	-1.140794
O	1.493628	0.186302	-0.021907
S	0.951820	0.902315	1.212636
O	-2.516514	-1.363427	-1.502320
O	2.361182	1.522685	1.617270

TS-BCR1BCR2

O	-0.131154	-0.262520	0.066397
S	0.305430	0.414410	1.378341
O	1.202990	-0.287561	2.363980
H	2.621409	-1.385680	1.978743
O	3.365444	-2.007083	1.815291
N	3.092934	-2.553165	0.566495
O	3.887776	-3.363351	0.191519
O	2.093636	-2.149466	0.003530

TS-BCR2BCR3

O	0.000147	-0.003583	-0.001262
N	-0.001451	0.000151	1.221401
O	0.938846	0.001791	1.957146
O	-1.232248	0.002843	1.841906
O	-3.032849	-0.002443	-0.245190
S	-2.349180	-0.006428	-1.586784
O	-3.446928	-0.008684	-2.752200
H	-1.894061	0.000992	1.105300

BTS1

O	-0.038946	0.259031	0.015545
S	0.012265	0.096143	1.561673
O	1.423485	-0.372477	1.980112
H	2.320696	-0.052879	1.177112
O	3.214376	0.245139	0.486790
N	2.969446	-0.024607	-0.751362
O	3.846717	0.004936	-1.569511
O	1.784595	-0.328339	-1.097044

BTS2

O	.022859	-.005418	-.071425
S	.026348	-.001424	1.432109
O	1.446705	.007964	2.071711
H	1.258068	.008543	3.273302
O	1.025899	.008900	4.455997
N	-.243426	.004073	4.670216
O	-.677512	.004117	5.787997
O	-1.033238	-.000831	3.672558

BTS3

N	-0.016171	-0.018540	0.002964
O	-0.019945	-0.297659	1.251852
O	1.131261	0.229422	-0.534498
H	1.838454	0.096487	0.397183
O	2.203085	-0.181044	1.550033
S	2.767896	1.028413	2.504858
O	-1.035003	0.004438	-0.619893
O	1.657311	1.834617	3.076066

BTS4

O	2.051877	1.130422	-0.651495
S	2.172676	-0.087075	0.230428
O	1.019320	-1.131250	0.140520
H	-0.127952	-0.811274	-0.251792
O	-1.162499	-0.649542	-0.720644
N	-1.930163	0.096415	0.058505
O	-3.048442	0.284482	-0.385191
O	-1.499824	0.523207	1.104555

BTS5

N	0.065800	-0.039164	-0.013868
O	0.079639	-0.072553	1.268582
O	1.178185	0.244076	-0.598749
H	1.896860	0.364280	0.343730
O	2.266860	0.323970	1.512982
S	2.408492	1.766635	2.320926
O	-0.940803	-0.260602	-0.617481
O	3.581435	2.443984	1.699965

BTS6

N	0.005812	0.011897	-0.004778
O	0.032963	-0.023116	1.209935
O	0.962585	0.037715	-0.744623
O	-1.220649	0.028968	-0.514138
H	-1.194494	-0.027510	-1.678719
O	-1.347899	-0.127661	-2.892125
S	-0.847868	1.044530	-3.822685
O	-0.711727	0.280045	-5.138014

BCP1

O	1.665268	-0.087400	-0.685180
S	1.083783	0.044621	-2.048878
O	-0.556332	-0.122056	-1.952256
H	-0.842344	0.043284	-1.036846
O	-0.891766	0.388653	1.094230
N	0.011985	0.039084	1.834099
O	0.756940	0.856489	2.392070
O	0.234735	-1.151647	2.088768

Table S11. Cartesian coordinates (in Angstroms) of the stationary points for the reaction of $^3B_1$ of SO_2 with H_2O, computed at B3LYP/aug-cc-pVTZ level of theory.

CCR1

O	-0.738917	0.016690	-0.953915
S	-0.774254	0.018706	0.648640
O	0.630507	0.030182	1.194143
O	1.460876	-0.130190	-1.391006
H	1.559134	-0.039590	-0.418527

H	1.396306	0.768601	-1.738418

CTS1

O	0.000735	-0.001530	-0.005862
S	-0.002904	0.001337	1.538490
O	1.501664	-0.000431	1.969320
O	2.199308	-0.142066	-0.317932
H	2.040798	-0.031434	0.911765
H	2.066574	0.716838	-0.748468

CCP1

O	-0.831077	0.036699	-0.876421
S	-0.826322	0.029202	0.624274
O	0.721505	0.009179	1.130288
O	1.617907	-0.117083	-1.400349
H	1.293403	0.017788	0.317556
H	1.166484	0.565613	-1.928069

CCR2

O	1.937425	0.212725	0.051203
S	0.573365	-0.410737	-0.030471
O	-0.349801	0.919285	0.064026
O	-2.426458	-0.309559	-0.098905
H	-2.600633	0.297153	-0.828042
H	-2.644175	0.187250	0.698515

CTS2

O	0.408424	0.145769	-0.153168
S	0.176365	0.105441	1.329772
O	1.484177	-0.480640	2.060655
H	2.453061	-0.381216	1.358152
O	3.023749	-0.631105	0.375465
H	2.474590	-0.236764	-0.333700

CTS3

O	-0.248156	0.063157	0.009997
S	0.000018	-0.006728	1.475795
O	1.585774	-0.003543	1.829315
H	2.287132	-0.628483	1.142712
O	2.895754	-0.827711	0.145374
H	3.499844	-0.075206	0.033983

CTS4

O	1.986548	0.283304	-0.140429
S	0.710046	-0.449985	0.066138
O	-0.426016	0.754900	0.104100
O	-2.451795	-0.217815	-0.173505
H	-1.432123	0.423434	-0.307207
H	-2.908804	0.163247	0.594185

CCP2

O	0.052185	0.446686	-1.243888
S	0.883653	0.271346	-0.010996
O	-0.101673	0.063996	1.279080
O	-2.526146	0.111575	-0.137719
H	-1.042905	0.036215	0.978788
H	-1.895345	0.258845	-0.881943

CCR3

O	0.060057	-0.012325	-0.035853
O	0.125010	-0.237783	2.798565
S	1.603342	0.093477	2.414384
O	2.094692	0.027113	3.873659
H	-0.436151	0.799422	-0.184493
H	-0.600149	-0.666358	0.217898

TS-CCR2CCR3

O	0.012786	0.001930	-0.000492
O	0.107346	-0.001560	2.655693
S	1.675631	-0.000531	2.419085
O	2.045030	-0.002513	3.895441
H	-0.562707	0.766426	0.112463
H	-0.563285	-0.762205	0.111969

CR4

S	0.307591	-0.563460	0.142221
O	-0.318755	0.274193	1.292051
O	1.508607	0.400622	-0.024275
H	3.261287	-0.222988	1.063456
O	3.818510	-0.925231	1.419381
H	4.481041	-0.479938	1.954976

TS-CCR3CCR4

S	-0.026244	0.056568	0.020996
O	0.017079	-0.000526	1.573169
O	1.516116	-0.008368	-0.113574
H	2.186319	-1.806243	-1.072122
O	1.955479	-2.472116	-1.730694
H	2.790211	-2.750515	-2.118136

CCR5

O	0.002668	-0.439106	0.055578
S	1.042133	-0.094291	1.107319
O	0.719557	0.558800	2.429235
O	3.853088	-0.397023	1.375319
H	3.997274	0.145403	2.158826
H	4.079646	-1.294714	1.641287

TS-CCR1CCR4

S	0.209518	0.440200	0.132425
O	-0.071930	-0.463429	1.433919
O	1.284733	-0.397318	-0.521946
H	2.086650	-1.927969	0.682315
O	2.273465	-2.499368	1.438376
H	1.751212	-3.293266	1.289235

TS-CCR3CCR5

S	-0.037170	-0.006548	0.032707
O	0.072482	0.022883	1.642295
O	1.334546	-0.007170	-0.570528
H	0.302761	-0.037856	-2.769289
O	-0.661511	-0.022512	-2.743783
H	-0.944666	-0.831139	-3.182392

References

(1) Anglada, J. M.; Martins-Costa, M. T. C.; Francisco, J. S.; Ruiz-López, M. F. Triplet State Promoted Reaction of SO2 with H2O by Competition Between Proton Coupled Electron Transfer (pcet) and Hydrogen Atom Transfer (hat) Processes. *Phys. Chem. Chem. Phys.* **2019**, *21*, 9779–9784. https://doi.org/10.1039/C9CP01105F.

(2) M. J. Frisch; J. A. Pople; J. S. Binkley. Self-Cons¡stent Molecular Orbital Methods 25: Supplementary Functions for Gaussian Basis Sets. *J Chem Phys* **1984**, *80*, 3265–3269.

(3) Hehre, W. J.; Ditchfield, R.; Pople, J. A. Self—Consistent Molecular Orbital Methods. XII. Further Extensions of Gaussian—Type Basis Sets for Use in Molecular Orbital Studies of Organic Molecules (6-31G* Basis Set). *J Chem Phys* **1972**, *56*, 2257–2261.

(4) T. H. Jr. Dunning. Gaussian Basis Sets for Use in Correlated Molecular Calculations. I. The Atoms Boron through Neon and Hydrogen. *J Chem Phys* **1989**, *90*, 1007.

(5) Kendall, R. A.; Dunning, T. H.; Harrison, R. J. Electron-Affinities of the 1St-Row Atoms Revisited - Systematic Basis-Sets and Wave-Functions. *J Chem Phys* **1992**, *96* (9), 6796–6806.

(6) Helgaker, T.; Klopper, W.; Koch, H.; Noga, J. Basis-Set Convergence of Correlated Calculations on Water. *J. Chem. Phys.* **1997**, *106* (23), 9639–9646. https://doi.org/10.1063/1.473863.

(7) Troe, J. Theory of Thermal Unimolecular Reactions at Low Pressures. II. Strong Collision Rate Constants. Applications. *J. Chem. Phys.* **1977**, *66* (11), 4758–4775. https://doi.org/10.1063/1.433838.

(8) Machado, G. de S.; Martins, E. M.; Baptista, L.; Bauerfeldt, G. F. Theoretical Investigation of the Formic Acid Decomposition Kinetics. *Int. J. Chem. Kinet.* **2020**, *52* (3), 188–196. https://doi.org/10.1002/kin.21341.

(9) J. Troe. Theory of Thermal Unimolecular Reactions at Low Pressures. I. Solutions of the Master Equation. II. Strong Collision Rate Constants. Applications. *J Chem Phys* **1977**, *66* (11), 4745–4775.

(10) Hippler, H.; Troe, J.; Wendelken, H. J. Collisional Deactivation of Vibrationally Highly Excited Polyatomic Molecules. II. Direct Observations for Excited Toluene. *J. Chem. Phys.* **1983**, *78* (11), 6709–6717. https://doi.org/10.1063/1.444670.

(11) Python Software Foundation. Python Language Reference, version 3 4 3 A. at

http://www python org. .
(12) S. W. Benson. *Thermochemical Kinetics*, 2nd ed.; Wiley-Interscience: New York, 1976.
(13) Anglada, J. M. Complex Mechanism of the Gas Phase Reaction between Formic Acid and Hydroxyl Radical. Proton Coupled Electron Transfer versus Radical Hydrogen Abstraction Mechanisms. *J Am ChemSoc* **2004**, *126* (31), 9809–9820.
